# Personalized Classification of Scenario-Derived Operational Driver-State Classes from Non-Intrusive Wearable Signals in Real-World SAE Level 2 Automated Driving

**DOI:** 10.3390/s26144529

**Published:** 2026-07-17

**Authors:** Raul Fernandez-Matellan, David Puertas-Ramirez, David Martin Gomez, Jesus G. Boticario

**Affiliations:** 1 Intelligent Systems Lab, Electrical Engineering Department, Universidad Carlos III de Madrid, 28911 Leganés, Spain; dmgomez@ing.uc3m.es; 2 aDeNu Research Group, Artificial Intelligence Department, Universidad Nacional de Educación a Distancia, 28040 Madrid, Spain; dpuertasr@dia.uned.es (D.P.-R.); jgb@dia.uned.es (J.G.B.)

**Keywords:** drivermonitoring systems, automated driving, wearable sensors, physiological signals, motion signals, personalized modeling, real-world driving, scenario-derived operational labels, multi-sensor acquisition

## Abstract

**Highlights:**

**What are the main findings?**

- A real-world SAE Level 2 setup was used to collect multi-sensor data during repeated on-road experiments.
- Target-driver-only training achieved higher Leave-One-Experience-Out performance than configurations incorporating external-user data.

**What are the implications of the main findings?**

- The results provide initial feasibility evidence for intra-subject classification of scenario-derived operational classes using wearable signals.
- The target-driver formulation provides a practical personalization route based on repeated individual-driver data and a basis for future deployment-oriented evaluation.

**Abstract:**

At SAE Level 2 automation, the human driver retains full supervisory responsibility, making unobtrusive monitoring relevant for maintaining supervision under real-world driving conditions. Driver monitoring systems capable of operating robustly under such conditions are therefore essential, but wearable-based personalized approaches remain underexplored, particularly when the target labels are derived from experimental scenarios. This study presents a real-world SAE Level 2 on-road acquisition campaign and evaluates a target-driver intra-subject classification approach using non-intrusive wrist-derived signals. Physiological and motion data recorded with the Empatica E4 wristband, including blood volume pulse, electrodermal activity, heart rate, skin temperature, and triaxial wrist acceleration, were converted into image representations and processed with a frozen ResNet-50 feature extractor, principal component analysis, and a supervised classifier. The labels were scenario-derived operational driver-state classes defined from experimental phases and scenario groups. Personalization was assessed via a Leave-One-Experience-Out protocol on the target driver. Classification accuracy was 50% under external-user-only training, 54% under mixed target/external-user training, and 60% under target-driver-only training, with the target-driver-only configuration yielding the highest mean performance in the evaluated setting. For the low-demand baseline class, the one-vs.-rest classifier achieved 88.4% accuracy and an F1-score of 70%. These results provide initial evidence of the feasibility of personalized wrist-worn classification of scenario-derived operational driver-state classes under the real-world automated driving conditions evaluated in this study.

## 1. Introduction

The increasing deployment of driving automation functions has created new opportunities for safety, comfort, and driver assistance. At the same time, it has reinforced the need to understand human–automation interaction during assisted and automated driving [1,2] and monitor driver state under these conditions [3,4]. This concern is reflected in recent crash-reporting initiatives, such as the Standing General Order program of the U.S. National Highway Traffic Safety Administration, which collects real-world crash reports involving vehicles equipped with Automated Driving Systems (ADSs) and SAE Level 2 Advanced Driver Assistance Systems (ADASs) [5]. In this context, driver monitoring systems (DMSs) are becoming essential safety-oriented technologies for monitoring driver condition during assisted and partially automated driving. At SAE Level 2, the Driving Automation System (DAS) can provide sustained lateral and longitudinal vehicle motion control, yet it performs only part of the Dynamic Driving Task (DDT) [6]. The human driver remains responsible for supervising the driving environment, performing Object and Event Detection and Response (OEDR), and resuming manual control whenever system limitations or road conditions require intervention [6]. This allocation of responsibilities is central to the present work because the study focuses on driver monitoring during partially automated driving, where the driver must remain engaged despite sustained automation support. This framework is consistent with ISO/TR 21959-1:2020, which addresses human performance and driver-state concepts in the context of automated driving functions that still require human participation, continuous supervision, or readiness to intervene [7]. At the vehicle-system level, recent work has investigated V2X-assisted distributed computing and cooperative control for connected and automated vehicles in complex merging scenarios [8].

The work presented here focuses specifically on wearable-signal classification of scenario-derived operational conditions under partially automated driving conditions in the real world. Simulator and controlled-environment studies have provided valuable evidence because they allow repeatable, safe, and well-controlled experimental conditions [9,10,11]. Real-world driving, however, introduces additional challenges: road variability, traffic dynamics, motion artifacts, sensor placement constraints, and imperfect signal quality. These challenges are especially relevant for DMSs intended to operate outside the laboratory, where sensing devices must be robust, acceptable to the user, and compatible with future vehicle integration. Recent studies confirm the growing interest in wearable and non-intrusive sensing for driver monitoring. Recent examples include smartwatch-based prediction of driver state and takeover performance [12], wearable systems for monitoring driver motion and physiological condition [13,14], and cross-subject modeling of situation awareness in automated driving [15]. Among the available sensing modalities, wrist-derived physiological and motion signals offer a practical compromise, as they can capture cardiovascular activity, electrodermal activity, skin temperature, and body motion with limited interference in driving tasks [16,17,18]. For this reason, the present study uses the Empatica E4 wristband, a research-grade wearable device commonly used in ambulatory settings to record blood volume pulse (BVP), electrodermal activity (EDA), skin temperature (TEMP), and triaxial acceleration, together with heart rate (HR) estimates derived from the photoplethysmography (PPG)-based BVP signal [19,20].

A second key challenge concerns personalization. Many driver-state modeling approaches are designed to generalize across users, but physiological and behavioral responses are highly individual and may vary across sessions, road contexts, and sensor conditions [16,18,21]. A model trained to represent an average driver may therefore fail to capture stable within-driver patterns that are relevant for personalized monitoring. To address this, the present study adopts an intensive longitudinal target-driver design. Rather than treating the primary driver as a small sample of a population, repeated observations from the same driver are used to assess whether a personalized intra-subject model can learn driver-specific patterns under real on-road SAE Level 2 automation. This rationale draws on N-of-1 and single-case longitudinal approaches, where within-person dynamics are characterized before broader population-level generalization is attempted [22,23]. Accordingly, this manuscript does not claim population-wide validity; instead, it evaluates an initial real-world feasibility step for personalized DMSs based on repeated data from a target driver. This design is further motivated by prior evidence that intra-subject models can be effective when physiological responses differ substantially between users [24,25,26].

Against this background, this paper evaluates target-driver intra-subject classification of scenario-derived operational driver-state classes using real-world data collected during SAE Level 2 automated driving. The acquisition setup records physiological, behavioral, vehicle-related, and environmental data during on-road experiments, while the modeling pipeline evaluated in this manuscript focuses on wrist-derived BVP, EDA, HR, skin temperature, and triaxial acceleration from the Empatica E4 device. The remaining sensor streams are used to support scenario verification, temporal annotation, contextual interpretation, synchronization, safety supervision, and future multimodal extensions. The target classes are scenario-derived operational driver-state classes. These labels are obtained from predefined experimental phases and scenario groups rather than from validated psychological scales. Examples include parked baseline relaxation, manual driving, sustained DAS engagement with scripted gaze or head-orientation tasks, and higher-demand events such as lane changes or sudden acoustic startle scenarios. The central research question is therefore whether non-intrusive wearable signals can discriminate between experimentally defined driving conditions for a target driver under real-world Level 2 automation.

The experimental evaluation is guided by the following hypotheses:

**Hypothesis** **1.** *Non-intrusive wrist-derived physiological and motion signals contain sufficient information to discriminate these scenario-derived operational driver-state classes for a target driver under real-world SAE Level 2 automated driving conditions.*

**Hypothesis** **2.** *In the evaluated primary target-driver setting, intra-subject training provides better session-level classification performance than configurations that incorporate the available external-user data.*

To examine these hypotheses, we compare training configurations based on target-driver data, external-user data, and combinations of both. The acquisition campaign collected 26 driving sessions from the primary target driver, corresponding to approximately 13–19.5 h of recorded driving. Data from the remaining users, collected in fewer sessions, are used as complementary external-user data to assess whether non-target-user variability helps or degrades target-driver classification in the same evaluation setting. The primary evaluation uses a Leave-One-Experience-Out (LOEO) protocol, which assesses session-level generalization and reduces the risk of temporal leakage between highly correlated sliding windows. Random sampling (RS) and ablation analyses are also reported, but they are treated as development-oriented experiments for studying signal selection, dimensionality reduction, and signal-to-image encoding choices.

The main contribution of this work is an initial real-world feasibility study of target-driver intra-subject classification of scenario-derived operational driver-state classes using non-intrusive wearable signals under SAE Level 2 automated driving. More specifically, this work contributes:

- A real-world on-road data acquisition study for driver monitoring under SAE Level 2 automated driving, encompassing wearable sensing, onboard cameras, depth sensing, synchronization procedures, and safety supervision.
- A dataset construction procedure based on wrist-derived Empatica E4 signals, including BVP, EDA, HR, skin temperature, and triaxial acceleration, together with scenario-derived operational driver-state classes obtained from predefined experimental phases and driving scenarios.
- A target-driver feasibility evaluation of whether non-intrusive wearable physiological and motion signals can discriminate operational driver-state classes in real-world SAE Level 2 automated driving.
- A systematic comparison of target-driver-only, external-user-only, and mixed target/external-user training configurations, with LOEO used as the main session-level evaluation protocol.
- An empirical assessment of methodological choices relevant to low-data personalized DMS research, covering signal-to-image encoding, wearable-signal selection, feature-level fusion, dimensionality reduction, and dataset partitioning.

The paper is organized as follows. Section 2 reviews prior work on driver monitoring, real-world experimentation, wearable sensing, personalization, and signal-to-image learning. Section 3 presents the real-world multi-sensor acquisition setup. Section 4 describes the experimental protocol, scenario execution, and safety supervision. Section 5 explains dataset construction and scenario-derived labeling. Section 6 details the wearable-signal modeling pipeline, including signal-to-image encoding, feature extraction, feature-level fusion, dimensionality reduction, classification, training configuration, and dataset partitioning. Section 7 reports the primary LOEO evaluation and exploratory ablation analyses. Section 8 discusses interpretation, limitations, and deployment implications. Section 9 and Section 10 summarize conclusions and future research directions.

## 2. Related Work

As automation advances and reduces the tasks required of users, staying focused becomes more challenging, making it difficult to maintain situational awareness [27]. This issue is particularly significant at SAE Levels 2 and 3 [28,29], although the driver’s role differs across these automation levels. At SAE Level 2, the driver must continuously supervise the DAS and the driving environment [6], whereas Level 3 work more commonly emphasizes fallback readiness and takeover requests [29]. In safety terms, the goal is to reduce supervision failures and unsafe responses during unexpected events or when exiting the intended Operational Design Domain (ODD) [1]. One approach is to model the driver’s state and provide feedback to help maintain an adequate supervisory condition while in the vehicle. However, this topic is complex due to the inherent diversity of individual users and situations. Consequently, there are multiple approaches to user modeling. Some researchers focus on identifying aspects related to the user’s awareness such as attention [30], drowsiness [31], fatigue [32], or distraction [33]. Recent deep attention-based architectures have also been proposed for driver-distraction detection [34]. Recent architectures also address driver-state recognition more broadly [4]. Temporal architectures have also been applied to driver-fatigue recognition under real-road conditions [35]. Other studies examine the human operator’s relationship with the DAS, examining how drivers engage with non-driving-related tasks [36] and emphasizing aspects such as acceptance [37], workload [38,39], and trust [40]. The reviewed studies define the conceptual context for this manuscript. They show that driver-state research has been approached through related but distinct constructs, including attention, drowsiness, fatigue, distraction, workload, trust, acceptance, and situation awareness. Because these constructs differ in definition and measurement, the present study uses them as background for the monitoring problem while defining the supervised labels directly from the on-road protocol. Specifically, the proposed pipeline processes wrist-derived physiological and motion streams and classifies four *scenario-derived operational driver-state classes* obtained from predefined experimental phases and scenario groups. This formulation keeps the learning task tied to reproducible protocol events and clarifies the scope of the results: the study evaluates whether repeated wearable recordings from a target driver can discriminate those operational classes under real-world SAE Level 2 driving conditions.

This variety of user-modeling approaches also correlates with a diversity of experimental methodologies and environments. Most authors opt to perform tests in simulation environments, as this way the complete environment can be controlled and dangerous situations can be tested safely [9], with varying degrees of realism and immersion. Simulator studies are especially valuable for takeover-performance analysis [11], mental-state assessment [10], and controlled manipulation of automation-related scenarios. While simulations provide valuable insights and a controlled environment for testing, they have limitations and may not fully capture the intricacies, challenges, and unexpected situations inherent in real-world data, which are essential for gaining a deeper understanding of the problem [41]. This distinction is important for ensuring driver safety under operational conditions. In these situations, context and human responses may not always align with laboratory assumptions. DMS plays a crucial role in monitoring the driver’s state during periods of automation. Real-world and on-road studies are therefore necessary to test whether driver monitoring approaches remain useful under practical sensing conditions, where traffic context, road variability, body motion, sensor placement, and synchronization constraints can affect the recorded signals [17]. The present study follows this direction by collecting on-road data during SAE Level 2 automated driving, where scenario execution, safety supervision, and signal quality must be handled outside the laboratory.

Regardless of the driver state of interest, sensor selection remains an open issue, with options including physiological sensing, visual sensing, or combinations of both [18,42]. Driver-state sensing can also be supported by driver-facing cameras [42], eye-tracking or visual–behavioral measures [33], vehicle and steering signals [43], physiological wearables [16], and multimodal combinations [18]. In the present acquisition setup, camera streams were recorded and used for protocol verification, temporal annotation, contextual interpretation, and quality control. However, the modeling pipeline evaluated in this manuscript focuses on wrist-derived physiological and motion signals in order to assess their contribution independently from camera-based features. The general consensus is that a multimodal approach yields the most accurate models and the best results, given the complexity and individual variations among users [28]. The selection of suitable physiological sensors is particularly challenging due to the diversity of signal types and applications [16]. In laboratory simulations, researchers often opt for “intrusive” sensors because they provide more reliable readings [10,44]. Electroencephalography (EEG) and electrocardiography (ECG) are valuable tools for assessing cognitive load, fatigue, drowsiness, and takeover readiness, particularly in controlled experiments, validation-oriented protocols, and studies aimed at characterizing specific physiological mechanisms. In the present study, however, the target sensing profile is lower-intrusion and repeatedly deployable during on-road SAE Level 2 sessions. Electrode-based systems generally necessitate meticulous placement, calibration, signal-quality control, and user acceptance management, making them a more intrusive configuration than the one evaluated here [45,46,47]. Their results are therefore useful as complementary evidence for DMS research, while the experimental pipeline evaluated in this manuscript prioritizes a wearable configuration with lower setup burden.

For this reason, recent driver-monitoring studies increasingly consider wearable and low-intrusion devices as a practical alternative for real-world or in-the-wild data collection [17]. At the lowest end of the intrusion spectrum, remote physiological sensing based on cameras or radar is especially attractive because it can estimate vital signs without body contact. Contactless eye-blink monitoring has also been demonstrated using radar-based sensing in real time [48]. Camera-based remote photoplethysmography (rPPG), for example, has recently been explored for driver monitoring, but current studies still report open challenges related to illumination changes, head motion, vehicle vibrations, dataset diversity, and cross-dataset generalization [49,50]. In this context, wrist-worn sensing provides a practical intermediate option between electrode-based acquisition and fully remote physiological monitoring: it is less direct than EEG or ECG and can still be affected by motion artifacts, but it can capture cardiovascular activity, electrodermal activity, skin temperature, and body motion with limited interference in the driving task [18].

Wrist-worn devices are especially relevant in this context because they can capture cardiovascular activity, electrodermal activity, skin temperature, and body motion with limited interference in the driving task [18]. Recent work has also explored smartwatch-based driver-state prediction [12], wearable monitoring of driver motion and physiological condition [13], and low-cost sensing for driver monitoring [14]. Although wrist-derived signals can be affected by motion artifacts and sensor placement, the Empatica E4 has been used and validated as a research-grade wearable device for ambulatory physiological monitoring, supporting its selection in this work [19,20].

In terms of modeling techniques, personalized driver-state sensing faces a practical constraint: collecting large amounts of labeled data from each driver is difficult, especially in real-world automated driving. Methods that reuse existing feature extractors or transform short time windows into compact representations are therefore well suited to low-data settings. A common approach is to convert 1D physiological data into 2D representations. This strategy has been adopted in physiological signal classification [51] and driving-related signal analysis [52]. Techniques such as Markov Transition Fields (MTFs) [53], Gramian Angular Fields (GAFs) [54], and Recurrence Plots (RPs) [55] can be used for this purpose. In this work, RPs are used to capture temporal recurrence, GAFs to encode angular temporal correlations, and MTFs to describe transition structure. Because labeled data are scarce relative to the available unlabeled physiological recordings, this work incorporates an unsupervised, configuration-specific representation calibration step based on Principal Component Analysis (PCA). This is a label-efficient strategy because unlabeled recordings are used to estimate a compact feature space, while supervised classifier training remains restricted to labeled operational classes [56]. Empirical comparisons of RPs, GAFs, and MTFs are conducted with a methodological purpose: to identify which signal-to-image representation is best suited to the proposed wearable-signal pipeline, without claiming that image encoding is universally superior to handcrafted physiological features or temporal models.

### Personalized Models

Previous research has predominantly focused on developing generalized (inter-subject) models. For example, one study evaluated driver readiness to take over using data from 81 participants in a driving simulator [57]; similarly, another used 80 participants with repeated takeover events [58], and another examined takeover behavior in 20 subjects [59]. These studies are valuable because they identify population-level factors and support broader conclusions about driver behavior. However, such inter-subject approaches often struggle to capture individual differences in affective or cognitive states. One study reported an average accuracy of 74% across 19 users, but individual results varied widely from nearly 90% to as low as 34.7% [21]. This variability is important for DMSs because a model that performs reasonably well on average may still fail to represent the stable physiological and behavioral patterns of a specific driver. This highlights a key limitation: generalized models may fail to account for the nuanced, person-specific patterns in driver behavior.

Personalized models have demonstrated significant performance gains across different fields. In emotion recognition using consumer wearable devices, one study reported 95.1% accuracy and a 91.7% F1-score using subject-specific models, while generalized counterparts performed markedly worse (67% accuracy, 43% F1) [26]. Comparable advantages of intra-subject modeling have been reported in education [25] and medicine [60]. Collectively, these works support the view that subject-specific physiological modeling is a credible methodological direction when individual variability is high and repeated labeled data can be collected from the same person.

Within automated driving, personalized approaches have improved drowsiness detection [61] and takeover readiness prediction, where models refined with subject-specific data outperform population-based baselines [62]. Some researchers have explored hybrid strategies, adapting generalized models using demographic or behavioral attributes, such as age or gender [63]. Despite their importance for safer DMS, personalized models are still underutilized due to deployment complexity. This deployment complexity includes the need for sufficient target-driver data, calibration across sessions, privacy protection, and model updating. This study addresses that gap by building a target-driver intra-subject modeling pipeline in a real driving environment using a non-intrusive data acquisition setup.

Recent automated driving studies further support the need to consider driver-specific information when modeling supervisory behavior and readiness. In SAE Level 2 automated driving, the TD2D dataset was designed to study takeover performance under secondary-task conditions. It includes physiological, ocular, workload, and takeover-performance data from 50 drivers. The authors also note that subject-independent models can support cold-start operation but may later be refined into subject-dependent models as driver-specific data accumulate [64]. Physiological responses have also been analyzed as indicators of takeover readiness in conditionally automated driving [11]. Recent ECG-based cognitive load classification work reported substantially higher performance in within-subject than across-subject evaluations [65]. These findings are consistent with previous takeover-readiness studies that examined the value of personalized versus general models [62], as well as recent work on personalized takeover prediction and driver-specific adaptation [59,63]. The present study builds on this line of work by evaluating a target-driver, within-subject setting in real-world, SAE Level 2 automated driving. Instead of treating the available participants as a population-level cohort, repeated observations are taken from the same drivers.

## 3. Data Acquisition

This section describes the real-world multi-sensor acquisition platform used in this study. The setup was designed to collect physiological, vehicle-related, and environmental data during SAE Level 2 automated driving for subsequent offline driver-state modeling.

### 3.1. Experimental Vehicle

Data acquisition was conducted using a Toyota Prius Plug-in Hybrid Electric Vehicle (PHEV), categorized as a conventional vehicle according to SAE J3016 (2021) [6]. Driving automation functionality was provided by the open-source Comma OpenPilot software, which enables Level 2 driving automation features in supported vehicles. Specifically, OpenPilot performs Adaptive Cruise Control (ACC) and Automated Lane Centering (ALC), delivering sustained lateral and longitudinal vehicle motion control.

The SAE Level 2 functionality used in this study was not developed as a new driving automation system by the authors. It was provided by the OpenPilot software running on Comma Three hardware. The authors configured and instrumented this platform for research data acquisition. All experiments were conducted with OpenPilot version 0.9.1. The vehicle’s native systems were left in the standard configuration required for normal OpenPilot operation. No additional modifications were made by the authors to disable, bypass, or alter the vehicle’s native ACC or lane-keeping functions beyond the standard OpenPilot installation and use.

OpenPilot operates by interfacing with the vehicle’s Controller Area Network (CAN) bus and transmitting control-related messages to the vehicle actuators. As an open-source platform developed on GitHub, OpenPilot provides access to internal variables and supports raw data logging for research purposes.

Comma OpenPilot also includes a camera-based driver-monitoring function that estimates driver head pose and facial orientation. In the present study, this function was part of the deployed SAE Level 2 automation stack, but its internal processed outputs were not used either as labels or as input features for the modeling pipeline. Only the recorded camera streams, vehicle-related logs, and wearable signals were retained for offline analysis. The OpenPilot driver-monitoring function should therefore not be interpreted as the DMS evaluated in this manuscript; the present work evaluates an offline wearable-signal modeling pipeline for personalized driver-state sensing.

### 3.2. Multi-Sensor Acquisition Setup

A multi-sensor acquisition platform was implemented to collect physiological, behavioral, vehicle-related, and environmental data during real-world driving. In line with Hypothesis 1, the modeling pipeline evaluated in this manuscript uses the wrist-derived Empatica E4 signals, while the remaining streams support protocol verification, contextual interpretation, and offline review. Table 1 summarizes the recorded streams.

The system consisted of the following components:

- **Body camera:** An *Intel RealSense D435i* was mounted inside the vehicle cabin to capture RGB video (1920 × 1080 at 30 fps), infrared images (1280 × 800 at 30 fps), and depth data (1280 × 720 at 30 fps). The camera was positioned to monitor the driver’s upper-body posture and movement.
- **Environmental camera:** The front-facing camera integrated into the Comma OpenPilot system was used to record the road scene. Video was captured at 10 fps and stored as raw image data without relying on OpenPilot’s processed outputs.
- **Face camera:** The infrared camera integrated into the Comma OpenPilot system was used to record the driver’s face at 10 fps. Only the raw infrared stream was used; no internal processing from OpenPilot was utilized.
- **Vehicle inertial measurement unit (IMU):** The IMU integrated into the Intel RealSense D435i was mounted on the vehicle to record acceleration and angular velocity.
- **Physiological signals—*****Empatica E4*** **wristband:** The driver wore an Empatica E4 wristband that recorded physiological data at the original sampling frequencies provided by the device. The following signals were captured: BVP at 64 Hz, HR at 1 Hz, EDA at 4 Hz, and skin temperature (TEMP) at 4 Hz. Data were streamed via Bluetooth and stored without modification in raw form for later analysis.
- **Wrist IMU—*****Empatica E4*** **wristband:** The same wristband also includes a 3-axis accelerometer, which recorded motion data at 32 Hz. This sensor was used to monitor the driver’s gross motor activity during vehicle operation.

**Figure 1:**
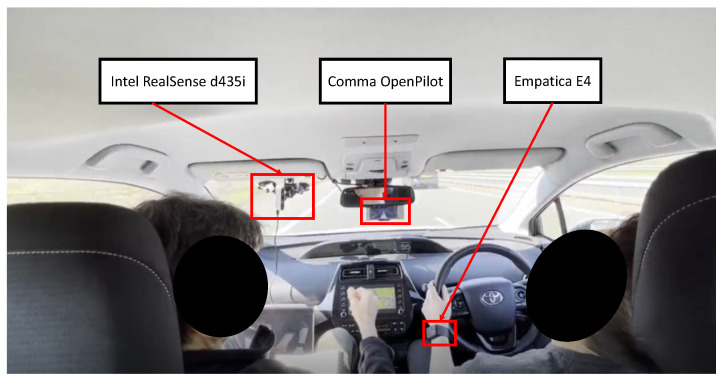
Sensor placement inside the experimental vehicle: Intel RealSense D435i, Comma OpenPilot device, and Empatica E4 wristband.

Sensor locations within the vehicle are shown in Figure 1. The Intel RealSense D435i was mounted on the windshield, on the passenger side, in a position that did not obstruct the driver’s field of view and allowed clear capture of the driver’s upper-body posture and movement. The Comma OpenPilot’s built-in environmental and infrared face cameras were positioned according to the system’s default configuration, facing forward and toward the driver’s face, respectively. The Empatica E4 wristband was worn on the participant’s non-dominant hand, placed approximately two fingers above the wrist joint, following the manufacturer’s recommended position to ensure proper physiological signal acquisition. All sensors were used with their default factory settings; no additional calibration procedures were required.

### 3.3. Operational Driving Conditions

All experiments were conducted in a consistent scenario involving two types of roadway environments:

1. **Manual-driving road segment:** A section where the DAS could not be engaged.
2. **Highways:** Road segments where the DAS could be engaged and operate according to its defined capabilities.

All tests were performed during the daytime under natural lighting conditions. Weather conditions were limited to clear or partly cloudy days; no experiments were conducted in fog, rain, or snow. Although traffic density could not be directly controlled, trials were scheduled outside of peak hours to ensure safety and minimize variability. Drivers were instructed to comply with posted speed limits throughout the route.

### 3.4. Timestamp Synchronization

Three independent time references were used during data collection: the Empatica E4 wristband, the host computer connected to the Intel RealSense D435i, and the Comma OpenPilot system. Each device maintains its own internal clock, and no centralized time synchronization protocol was implemented during recording.

To align data streams post hoc, a manual synchronization event was used. At the beginning of each session, the Empatica E4 event button was pressed while the action was simultaneously visible in the cabin video streams, providing a common visual and device-recorded reference for temporal alignment during post-processing. While this method provides sufficient synchronization for the analyses presented, the lack of a shared global timestamp across all devices introduces complexity. This procedure was repeated at the start of every recording for each participant and each session. Synchronization quality was assessed during offline review by checking the consistency between this initial synchronization event, the experiment notes, visible scenario transitions, and the temporal ordering of the aligned streams. Importantly, the supervised modeling pipeline uses only Empatica E4 signals as input features; these streams share the same device-level time reference and were resampled within that common temporal frame before image generation. The cross-device alignment was therefore mainly required for protocol verification, interval annotation, and contextual review, rather than for fusing asynchronous sensor streams in the classifier. Because each session was short and re-synchronized at its start, residual intra-session clock drift was considered acceptable for the reported analyses. Intervals for which synchronization or scenario identification was uncertain were excluded from the labeled dataset during quality control. Future work will consider integrating all sensors into a unified network with a common time base, which would simplify and automate the synchronization process and improve overall system efficiency.

### 3.5. Data Storage, Privacy, and Data Governance

Data from each sensor were stored using different systems according to device specifications. Information from Comma OpenPilot—including vehicle logs, face camera data, and environmental camera data—was stored locally on the Comma Three device. Data from the Empatica E4 wristband were uploaded to the Empatica cloud platform via Bluetooth. The Intel RealSense D435i data, including depth, infrared, RGB video, and IMU measurements, were recorded directly to an external hard drive during the experiments.

After each experimental session, all data were transferred to the institutional university cloud storage system. Data handling and storage complied with the General Data Protection Regulation (GDPR) and relevant institutional data protection policies.

Participant identifiers were replaced by pseudonymous user codes before analysis. Access to identifiable video, physiological data, and driving-context recordings was restricted to the research team. Because the dataset contains facial video, physiological signals, and real-world driving context, public release of the full dataset is not possible under the participants’ consent conditions and GDPR constraints. Data may be shared upon reasonable request, subject to institutional approval and data-sharing agreements.

### 3.6. Participants

The participants in this study were volunteers with prior experience using the instrumented vehicle and the OpenPilot-based SAE Level 2 system. This recruitment strategy was adopted because the study required repeated real-world driving sessions with an experimental acquisition setup, familiarity with the automation interface, and the ability to follow the safety and procedural requirements of the on-road protocol. No participant was recruited from the general population.

The experimental group included four male participants. Users 1, 2, and 4 were between 25 and 29 years old, while User 3 was between 45 and 49 years old. The sample composition reflects the available volunteer pool that met the operational requirements of the study. The objective of the analysis is not to compare demographic groups but to evaluate whether repeated data from a primary target driver can support personalized driver-state classification under real-world SAE Level 2 automated driving.

Prior to participation, each individual was fully informed about the nature and purpose of the data collection process, including how their data would be stored and used. Participants were explicitly informed of their right to withdraw from the study at any time, in which case their data would be permanently deleted. Informed consent was obtained from all participants, and all personal data were handled in compliance with applicable regulations in the local country and the European Union, as well as institutional ethical guidelines and best practices.

## 4. Experimentation

### 4.1. Experimental Protocol

**Figure 2:**
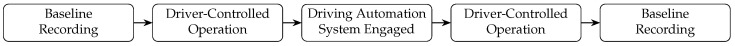
Experimental sequence showing transitions between manual and automated control phases.

All experimental sessions followed a common protocol consisting of five sequential phases: Baseline Recording, Manual Driving, DAS Engaged with scenario execution, Manual Driving, and a final Baseline Recording. This structure is illustrated in Figure 2. The protocol was designed to obtain repeated observations from the same target driver under comparable real-world driving conditions, while maintaining safety through continuous supervision by a co-driver.

**Baseline Recording:** The driver was asked to remain seated in a relaxed posture for three minutes with the vehicle parked in a safe location. This phase was intended to establish a low-demand reference period before driving. The driver was instructed to keep both hands relaxed, avoid unnecessary movements, and remain seated without interacting with the vehicle controls.

**Manual Driving:** From the parked state until highway entry, the vehicle was operated as a conventional vehicle. The driver was fully responsible for performing the DDT during this segment, as the DAS could not be engaged. During this phase, the driver was instructed to drive normally, comply with traffic regulations, and follow the route toward the highway segment where OpenPilot could be safely activated.

**DAS Engaged:** Upon reaching highway conditions that allowed engagement of the DAS, Comma OpenPilot was activated. During this phase, ACC and ALC provided sustained longitudinal and lateral vehicle motion control, respectively. During the DAS-engaged phase, the driver remained responsible for supervising the system and was instructed to resume manual control whenever required.

**Experiment Execution:** The co-driver was responsible for controlling the experiment flow, including starting and stopping recordings, measuring durations, and triggering each of the 16 scenarios in sequence. Whenever possible, these scenarios were conducted without disengaging Comma OpenPilot. However, in some cases, manual takeover by the driver was required due to system limitations or traffic conditions. The co-driver gave short verbal instructions immediately before each scenario. The purpose of these commands was to standardize the driver’s posture, gaze direction, hand position, or response condition during a short predefined interval. The 16 scenarios were executed sequentially, following the list and order detailed in Section 4.2, to preserve protocol reproducibility and traceability across sessions. The scenario order was therefore fixed rather than randomized, primarily to maintain safety, reproducibility, and ease of supervision during public-road experiments.

Each session lasted approximately 30–45 min, depending on real-world traffic conditions. As the experiments were conducted in naturalistic environments, factors such as traffic lights, highway access conditions, and congestion introduced some variability in timing. All sessions were conducted during daytime and under weather conditions considered safe for the protocol. If traffic density, road geometry, or system behavior prevented safe execution of a scenario, that scenario was delayed, shortened, or omitted from the valid labeled intervals.

### 4.2. Scenario Definitions

The following terms are used in the scenario descriptions. They correspond to the operational instructions given during the experiments and are defined to clarify the protocol:

- **Looking road:** the driver actively monitors the driving scene by scanning the forward roadway, surrounding traffic, mirrors, and relevant elements of the road environment.
- **No looking road:** the driver does not look at the driving scene and directs gaze away from the roadway and traffic environment.
- **Dashboard gaze:** the driver directs gaze toward the vehicle instrument cluster or speedometer during the predefined scenario interval. The head remains generally oriented forward, but the driver is not visually sampling the external road scene.
- **Road monitoring:** the driver maintains normal visual monitoring of the driving scene during the predefined scenario interval, including the forward roadway, surrounding vehicles, mirrors, traffic dynamics, and relevant events requiring supervision or possible intervention.
- **Head up:** the driver raises the head upward during the predefined scenario interval.
- **Head down:** the driver lowers the head downward during the predefined scenario interval.
- **Head turned to the opposite side:** the driver turns the head away from the side toward which the lane-change maneuver is being performed. For example, during a lane change toward the left, the driver turns the head toward the right side, and vice versa.
- **Hands on steering wheel/No hands on steering wheel:** the driver either keeps the hands in contact with the steering wheel or removes them during the predefined scenario interval.
- **Unexpected event:** a brief sudden acoustic stimulus produced by the co-driver during selected scenarios.
- **In no time:** the acoustic stimulus was delivered without prior warning during the corresponding scenario condition.

The predefined scenarios were:

1. **Lane change**, head turned to the opposite side, hands on steering wheel.
2. **Lane change**, head turned to the opposite side, **no** hands on steering wheel.
3. **Head up**, looking upwards raising his head, looking road, hands on steering wheel.
4. **Head up**, looking upwards raising his head, looking road, **no** hands on steering wheel.
5. **Head up**, looking upwards raising his head, **no** looking road, hands on steering wheel.
6. **Head up**, looking upwards raising his head, **no** looking road, **no** hands on steering wheel.
7. **Head down**, lowering his head but still looking road, hands on steering wheel.
8. **Head down**, lowering his head but still looking road, **no** hands on steering wheel.
9. **Head down**, lowering his head, **no** looking road, hands on steering wheel.
10. **Head down**, lowering his head, **no** looking road, **no** hands on steering wheel.
11. Looking road but **dashboard gaze** (not really looking at what is happening), hands on steering wheel.
12. Looking road but **dashboard gaze** (not really looking at what is happening), **no** hands on steering wheel.
13. Reaction to sudden startle (**unexpected event**) in no time during dashboard gaze, hands on steering wheel.
14. Reaction to sudden startle (**unexpected event**) in no time during dashboard gaze, **no** hands on steering wheel.
15. Reaction to sudden startle (**unexpected event**) in no time during road monitoring, hands on steering wheel.
16. Reaction to sudden startle (**unexpected event**) in no time during road monitoring, **no** hands on steering wheel.

To ensure consistency and reproducibility, the co-driver systematically administered each scenario and instructed the driver to perform the associated predefined action. For example, in Scenario 1, the co-driver prompted the driver with an instruction such as *“Lane change; turn your head away from the lane-change direction; keep your hands on the steering wheel.”*. This approach allowed us to standardize the enactment of each condition while maintaining safety; the co-driver continuously monitored the driving environment and road conditions throughout the experiment.

### 4.3. Safety Supervision

Because the experiments were conducted on public roads, safety constraints took priority over strict timing. The co-driver acted as experiment supervisor and safety monitor. The co-driver observed the road scene, surrounding traffic, automation behavior, driver posture, and scenario execution. The co-driver could interrupt a scenario, request the driver to resume normal monitoring, or request manual takeover whenever continuation was judged unsafe. Scenario intervals affected by interruptions, incorrect execution, missing signals, or synchronization problems were excluded from the final labeled dataset.

### 4.4. Recording Experiments

In total, data were collected across 39 driving sessions involving four participants: 26 sessions with User 1, 5 sessions with User 2, and 4 sessions each with Users 3 and 4. More than 2 terabytes of raw data were recorded, encompassing the full range of experimental conditions.

The dataset includes Comma OpenPilot log files containing recordings from two onboard cameras, namely, a forward-facing environmental camera and a driver-facing interior camera, as well as Intel RealSense D435i data comprising depth, infrared, RGB video, and IMU measurements. Physiological signals were primarily recorded using the Empatica E4 wristband, which forms the basis of the modeling pipeline described in this study. Several sessions also included data from additional wearable devices, such as EmotiBit and Fitbit wristbands, for exploratory complementary monitoring. These additional devices are not used in the analyses reported in this manuscript. They are mentioned only for completeness of the acquisition campaign, and their exclusion avoids mixing sensor types and preserves a consistent wrist-derived Empatica E4 input space across sessions.

## 5. Dataset Generation

This section describes how the recorded sessions were converted into the supervised dataset used by the modeling pipeline. The key point is that the labels are derived from experimental phases and predefined scenario groups. They are therefore referred to as *scenario-derived operational driver-state classes*. This terminology makes explicit that Low-Low (LL), Low (L), High (H), and High-High (HH) are operational class labels for supervised learning, not direct ground truth measurements of workload, distraction, stress, drowsiness, trust, or situation awareness.

### 5.1. Labeling Rationale

The labeling procedure was based on the predefined experimental protocol and the dataset-generation process summarized in Algorithm 1. The observational criteria described in multimodal affective studies [66] were used as a reference for offline visual review of protocol consistency. In particular, video recordings helped confirm that the requested observable action or phase had been correctly executed, that the selected interval matched the intended scenario or protocol phase, and that the relevant Empatica E4 streams were available. The supervised labels were assigned according to the protocol structure, scenario grouping, and interval-selection rules, yielding the scenario-derived operational classes introduced above.

The four-level classification scheme was designed to organize the experimental intervals into ordered operational categories with sufficient data coverage for supervised learning. The naming convention—LL, L, H, HH—is inspired by the ISA-18.2.5 alarm management standard [67] and the HL7 medical code system observation standard [68]. In this manuscript, these labels denote relative operational categories within the proposed protocol.

Four datasets were created, each corresponding to an individual driver. A total of 39 driving sessions were recorded: 26 with User 1, 5 with User 2, 4 with User 3, and 4 with User 4. This total includes all attempted real-world sessions, including sessions in which some scenarios were interrupted, incomplete, or affected by missing streams. For supervised dataset construction, only sessions in which the required Empatica E4 data were stored correctly and the protocol execution could be visually reviewed were retained. The final dataset includes 18 valid sessions: 9 from User 1, 4 from User 2, 3 from User 3, and 2 from User 4. Table 2 summarizes the recorded and retained sessions.

### 5.2. Operational Class Definitions

Each operational class is associated with a distinct phase or scenario group of the experiment. The classes are defined as follows:

- **Low-Low (LL)**: This class corresponds to the initial parked baseline recording, during which the driver was instructed to remain seated in a relaxed posture for three minutes. It is used as a low-demand reference interval within the protocol.
- **Low (L)**: This class corresponds to manual driving before DAS engagement, from the parked state to the highway segment where OpenPilot could be activated. During this interval, the driver performed the DDT manually under normal traffic conditions.
- **High (H)**: This class includes DAS-engaged scenarios involving scripted head orientation and gaze direction, namely, head up scenarios 3–6, head down scenarios 7–10, and dashboard gaze 11–12. These intervals represent moderate protocol-induced deviations from normal supervisory behavior.
- **High-High (HH)**: This class includes DAS-engaged scenarios involving lane-change maneuvers and sudden acoustic startle events, namely, scenarios 1–2 and 13–16. These intervals correspond to higher-demand operational events in the protocol.

Table 3 summarizes the mapping between the experimental protocol and the operational labels used for dataset generation.

The final baseline recording was retained for protocol completion and post-session contextual review, but it was not included in the supervised LL class in the analyses reported here. The LL class was constructed from the initial parked baseline to maintain a consistent pre-driving low-demand reference across sessions.

### 5.3. Interval Annotation and Quality Control

Each session was processed offline. First, the Empatica E4 signals were temporally aligned with the available video and contextual streams using the synchronization procedure described in Section 3.4. Then, the camera recordings and experiment notes were reviewed to identify the start and end of the baseline, manual driving, and scenario intervals. The purpose of this review was to verify protocol execution and interval boundaries, not to assign psychological or psychophysiological states from video. Once a valid interval was identified, its supervised label was determined by the predefined mapping between the experimental phase or scenario group and the corresponding operational class, as summarized in Algorithm 1.

Intervals were retained only when the corresponding Empatica E4 signal was available and the visual review confirmed that the intended phase or scenario could be identified. Sessions or intervals were excluded when required streams were missing, synchronization was not reliable, the baseline or scenario execution was incomplete, or the scenario had to be interrupted for safety or traffic-related reasons. These exclusions reflect the constraints of real-world on-road data collection, where traffic, system behavior, and sensor recording cannot be controlled as strictly as in a laboratory.

Each experimental session was processed following the procedure described in Algorithm 1, which enables the generation of labeled images used for training the models.
**Algorithm 1** Procedure for assigning scenario-derived operational driver-state classes. This method involves signal synchronization, quality verification, and annotation of valid time intervals that can be transformed into signal-encoded images for post-processing
1:Synchronize all sensors and signals.2:Analyze the experiment to detect and correct any errors. Common issues include: missing experiments, signals that cannot be synchronized, incorrect baseline recordings, or missing video or physiological signals.3:**if** the experiment is error-free **then**4:    Select and annotate the valid time periods corresponding to each operational class: **LL:** Initial baseline recording. **L:** Manual driving. **H:** scenarios involving Head Up (3, 4, 5, 6), Head Down (7, 8, 9, 10), and dashboard gaze (11, 12). **HH:** scenarios involving Reaction to sudden startle (13, 14, 15, 16) and Lane Change (1, 2).5:    Annotate each selected interval.6:    Apply Python scripts to transform these intervals into images.7:    Save each image in the folder corresponding to its label.8:**end if**

### 5.4. Generated Samples and Class Distribution

After image generation, the number of samples per user was as follows: 2158 for User 1, 1288 for User 2, 825 for User 3, and 754 for User 4. In this manuscript, one sample corresponds to one labeled time window after synchronization, annotation, and signal-to-image conversion. For each sample, seven Empatica E4 streams were represented: BVP, EDA, HR, TEMP, and the three wrist-acceleration axes (ACCEL_X, ACCEL_Y, and ACCEL_Z).

The number of samples generated for each class and user is presented in Table 4, and the corresponding percentage distribution in Table 5.

Although all participants followed the same protocol, the final number of samples differs across users because of session availability, valid interval duration, traffic-related timing differences, and exclusion of sessions or intervals affected by real-world recording constraints. These differences should not be interpreted only as a sampling limitation but also as an expected consequence of real-world target-driver data collection: even under the same protocol, each participant may generate different amounts of valid data because scenario execution, physiological responses, motion patterns, and signal quality vary across individuals and sessions. No class-level or participant-level balancing strategy was applied during dataset generation; therefore, Table 4 and Table 5 report the empirical distribution of valid labeled windows retained after synchronization, interval selection, and quality control. For the primary target driver, this empirical distribution presents a moderate class imbalance, with the L class accounting for 36% of the labeled windows, compared with 20–23% for LL, H, and HH. The distribution was retained in the reported experiments, and its potential influence is considered through the evaluation metrics described in Section 6.6 and the class-wise analysis presented in Section 7.2.

Image generation methods were also applied to auxiliary unlabeled segments, yielding 152,120 additional signal-encoded images. These images were derived from Empatica E4 recordings containing valid physiological and motion signals that could not be reliably assigned to any of the four operational classes due to incomplete scenario execution, missing contextual information, or other labeling constraints. Consequently, they carried no LL, L, H, or HH label, were excluded from supervised classifier training, and were never placed in supervised test folds as labeled samples. Their sole use was to fit the unsupervised PCA projection described in Section 6. Depending on the modeling configuration, this auxiliary unlabeled PCA set drew samples from the target driver, from external users, or both.

## 6. Wearable-Signal Modeling Pipeline

**Figure 3:**
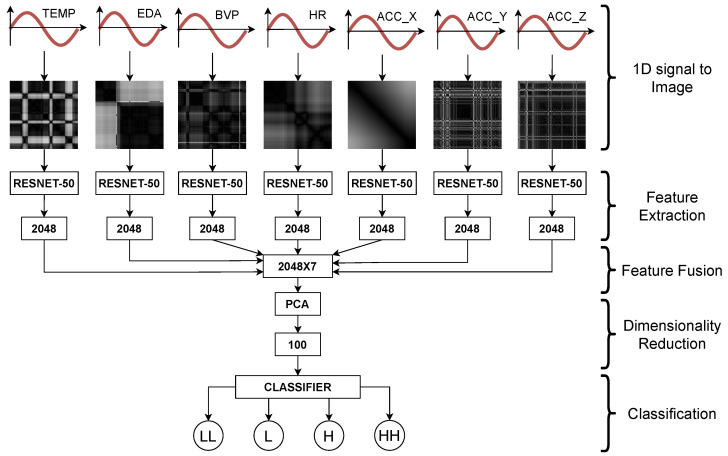
Overview of the offline wearable-signal modeling pipeline. Wrist-derived Empatica E4 signals are converted into image representations, processed by a frozen ResNet-50 feature extractor, fused at feature level, reduced using PCA and classified into the four scenario-derived operational classes.

This section describes the offline wearable-signal modeling pipeline applied to the labeled Empatica E4 windows generated in Section 5. Data acquisition, scenario execution, and label construction were described in Section 3, Section 4 and Section 5. The input to the pipeline is a 30 s window from seven wrist-derived streams: BVP, EDA, HR, TEMP, and triaxial acceleration. The output is one of the four scenario-derived operational labels defined in Section 5. The pipeline comprises resampling, signal-to-image encoding, ResNet-50 feature extraction, feature-level fusion, dimensionality reduction, and supervised classification, as summarized in Figure 3.

### 6.1. Windowing, Resampling, and Signal-to-Image Encoding

This subsection details the preprocessing steps and signal-to-image transformation techniques—specifically Recurrence Plots, Gramian Angular Fields, and Markov Transition Fields—used to generate physiological signal representations. It also outlines the parameter settings and the number of resulting images, which collectively define the input space for the downstream image-based feature-extraction pipeline.

#### 6.1.1. Signal Preprocessing

All Empatica E4 streams used by the modeling pipeline were resampled to a common frequency of 4 Hz using the scipy.signal module from the *SciPy* 1.11.3 Python library. The input streams correspond to the files exported from the Empatica Research Portal. BVP, EDA, TEMP, and triaxial acceleration were used as recorded by the device, whereas HR corresponds to the heart rate estimate provided by Empatica from the photoplethysmography/BVP signal [19]. Therefore, HR is treated as an Empatica-derived wearable stream rather than as a raw physiological waveform.

No additional handcrafted physiological features, artifact-removal algorithms, or frequency-domain transformations were applied before signal-to-image encoding. This decision was made to evaluate a simple and reproducible end-to-end representation pipeline in which temporal structure is preserved through the image encoding and feature extraction stages. We acknowledge that more specialized physiological preprocessing, such as motion-artifact correction, Heart Rate Variability (HRV) extraction, or Skin Conductance Response (SCR) decomposition, may improve performance, but such processing was outside the scope of the present feasibility study and is considered in the Section 8 and Section 10.

#### 6.1.2. Parameters for Signal-to-Image Generation

**Figure 4:**
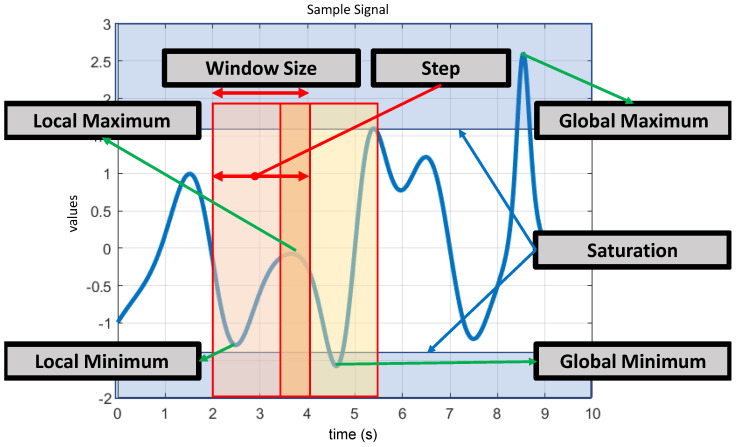
Visual representation of parameters for image generation from physiological signals: local/global maxima and minima, saturation values, working window width and step between consecutive images. The horizontal axis represents time in seconds.

The key parameters that influence the image generation process are illustrated in Figure 4.

- **Window Size:** Defines the time interval on the horizontal time axis for each generated image, set to 30 s in the experiments.
- **Step:** Represents the time shift along the same time axis between successive images, experimentally set to 3 s.
- **Scaling:** Ensures image values are in the range of [0, 255] using Equation (1).
  Three normalization methods were considered:
  – **Global Metrics:** Uses the entire signal for normalization, preserving scale but reducing contrast.
  – **Local Metrics:** Uses only the sliding window, enhancing contrast but losing relative scale.
  – **Combined Metrics:** This method, employed in our study, balances global and local normalization with parameter ϵ=0.5, formulated in Equation (2).
- **Saturation:** To mitigate noise and extreme values, signals are clipped at the 95th percentile (set to 255) and the 5th percentile (set to 0).

(1) $${\bar{x}}_{scale}^{i}=255\frac{x^{i}-\min_{x}}{\max_{x}-\min x}$$

(2) $${\bar{x}}_{end}^{i}=\epsilon \left({\bar{x}}_{global-metrics}^{i}\right)+\left(1-\epsilon \right)\left({\bar{x}}_{local-metrics}^{i}\right)$$

The windowing and scaling parameters were selected during preliminary development using training-side data and then kept fixed for all subsequent comparisons. Candidate configurations were assessed by checking whether the generated images preserved visible temporal structure, avoided excessive saturation, and produced stable input dimensions for the ResNet-50 feature extractor. No parameter was adjusted separately for a particular held-out session or evaluation result. This procedure was intended to keep the preprocessing pipeline fixed across experiments, so that performance differences could be attributed to the evaluated modeling configurations rather than to fold-specific preprocessing choices.

The following subsections summarize the three signal-to-image encodings evaluated in this work. Continuous RP was later selected for the main experiments because it achieved the best empirical performance in the encoding comparison reported in Section 7.

#### 6.1.3. Recurrence Plot (RP)

RP is a method for visualizing recurrent patterns in a trajectory $\vec{x}\in R^{d}$ within the phase space [55]. Equation (3) shows how a matrix (later visualized as an image) is generated, where *N* is the number of measured points, ϵ is a distance used as a threshold, Θ is the Heaviside function (beingΘ(x)=0ifx<0andΘ(x)=1otherwise), and ||·|| is the chosen norm to measure the distance.

For one-dimensional signals, $\vec{x}\in R$ and d=1; therefore, the RP matrix is constructed by computing pairwise differences between signal samples. By removing the constant ϵ and the Heaviside function Θ, we obtain the expression shown in Equation (4), which represents a continuous space (cont-RP) rather than a binary space (binary-RP), as would be the case in the original RP expression.

(3) $$R_{i,j}\left(\epsilon \right)=\Theta (\epsilon -||\vec{x_{i}}-\vec{x_{j}}||),\;i,j=1,\ldots ,N$$

(4) $$R_{i,j}=\left|\right|\vec{x_{i}}-\vec{x_{j}}\left|\right|,\;i,j=1,\ldots ,N$$

#### 6.1.4. Gramian Angular Field (GAF)

GAF is a technique for converting a signal into an image based on the assumption that the series *X* contains *N* elements $X=[x_{1},x_{2},\ldots ,x_{i},\ldots ,x_{N}]$ [54]. The algorithm is as follows:

1. Normalization is required to ensure that signal values fall within the appropriate range for the application of sinusoidal functions. In our approach, this is achieved through a scaling procedure that maps the values to the range of [−1,1], facilitating consistent input for downstream processing.
2. The one-dimensional time series from Cartesian coordinatesis converted to polar coordinates (see Equation (5)).
3. The angular summation and difference components are computed:
  - Gramian Angular Summation Field (GASF), see Equation (6).
  - Gramian Angular Difference Field (GADF), see Equation (7).

(5) $$\left\{\begin{array}{c} \phi_{i}=\arccos\left({\bar{x}}_{i}\right),\;-1\leq {\bar{x}}_{i}\leq 1,{\bar{x}}_{i}\in \bar{X}\\ r_{i}=\frac{i}{N},\;i\in N \end{array}\right.$$

(6) $$GASF=\left[\begin{array}{ccc} \cos(\phi_{1}+\phi_{1}) & \ldots & \cos(\phi_{1}+\phi_{n})\\ \cos(\phi_{2}+\phi_{1}) & \ldots & \cos(\phi_{2}+\phi_{n})\\ \vdots & \ddots & \vdots \\ \cos(\phi_{n}+\phi_{1}) & \ldots & \cos(\phi_{n}+\phi_{n}) \end{array}\right]$$

(7) $$GADF=\left[\begin{array}{ccc} \sin(\phi_{1}-\phi_{1}) & \ldots & \sin(\phi_{1}-\phi_{n})\\ \sin(\phi_{2}-\phi_{1}) & \ldots & \sin(\phi_{2}-\phi_{n})\\ \vdots & \ddots & \vdots \\ \sin(\phi_{n}-\phi_{1}) & \ldots & \sin(\phi_{n}-\phi_{n}) \end{array}\right]$$

#### 6.1.5. Markov Transition Fields (MTFs)

MTFs differ from previous methods because they use a probability-based approach to generate images from signals [53]. A state transition matrix is created to represent the probabilities of transitioning from one state to another and then these probabilities are used to create the images. The resulting image depends on the number of defined states. MTFs are based on the following considerations: There is a time series of the form $X=[x_{1},x_{2},\ldots ,x_{i},\ldots ,x_{N}]$, where the following states are defined: $Q=[q_{1},q_{2},\ldots ,q_{j},\ldots ,q_{Q}]$, and any point in the series $x_{i}$ can be associated with a state $q_{j}$. The implementation algorithm proceeds as follows:

1. Compute transitions between states *Q* using a first-order Markov chain. Then generate a matrix *W* of size Q×Q, where $w_{ij}$ is the probability that an element associated with the state $q_{j}$ is followed by an element associated with the state $q_{i}$. This matrix should accurately represent the probabilities of state transitions within the signal. The transition matrix can be generated either from the same time series that will be converted into an image or from a different time series that more accurately represents the transition probabilities between states.
2. Normalize the transition matrix so that $\sum_{j=1}^{Q}w_{ij}=1$.
3. Once the Markov transition matrix *W* is generated, the next step is to use it to generate the MTF. The MTF is a t×t matrix, where *t* is the length of the time series. To create the MTF, for each pair of time points $t_{i}$ and $t_{j}$ (where i,j=1,2,…,t), use the states that correspond to the values of the signal at those times. If the signal at time $t_{i}$ is associated with state $q_{a}$ and the signal at time $t_{j}$ is associated with state $q_{b}$, the value of the MTF at position (i,j) is set to the transition probability $w_{ab}$ from the Markov transition matrix *W*. See Equation (8).

(8) $$M_{ij}=\left[\begin{array}{ccc} w_{ij}|x_{1}\in q_{i},x_{1}\in q_{j} & \ldots & w_{ij}|x_{1}\in q_{i},x_{n}\in q_{j}\\ w_{ij}|x_{2}\in q_{i},x_{1}\in q_{j} & \ldots & w_{ij}|x_{2}\in q_{i},x_{n}\in q_{j}\\ \vdots & \ddots & \vdots \\ w_{ij}|x_{n}\in q_{i},x_{1}\in q_{j} & \ldots & w_{ij}|x_{n}\in q_{i},x_{n}\in q_{j} \end{array}\right]$$

### 6.2. Feature Extraction with Transfer Learning

Each image generated from an individual signal stream is passed through a Convolutional Neural Network (CNN) to extract relevant features. The core methodology uses transfer learning with ResNet-50 as a fixed feature extraction backbone.

Transfer learning uses a pre-trained neural network, such as ResNet-50 (which was originally trained on ImageNet), as a fixed feature extractor for related tasks. This leverages the network’s proven performance and generalization across computer vision applications. In this study, ResNet-50 is used as a fixed feature extractor for grayscale images generated from wrist-derived physiological and motion signals.

Features are extracted using all layers up to the global average pooling layer, with parameters frozen to preserve pre-trained knowledge without additional training. To accommodate ResNet-50’s 3-channel RGB input requirement, we replicate the single grayscale channel three times, yielding a 2048-dimensional feature vector per image. These vectors are then used as downstream feature representations and concatenated across wrist-derived signal streams during feature-level fusion. This enables the efficient and stable training of task-specific modules for driver-state analysis.

### 6.3. Feature-Level Fusion

This stage fuses information from multiple wrist-derived signal streams. Each image, derived from a different signal modality, is processed by the same frozen feature extractor (ResNet-50), which outputs a flattened feature vector. Specifically, ResNet-50 produces a 2048-dimensional vector per image. When using seven sensor modalities (BVP, EDA, HR, TEMP, ACCEL_X, ACCEL_Y, and ACCEL_Z), the resulting fused feature vectors have a dimensionality of 2048×7=14,336.

Feature extractors in our architecture are designed to receive 4-dimensional input tensors of shape [batch_size,channels,height,width]. However, due to the multimodal nature of our input (seven signals per sample), the input tensor has an additional modality dimension:

[batch_size,num_modalities,channels,height,width].

This 5D structure is incompatible with standard CNNs, which expect 4D input. Therefore, we reshape the input tensor before feature extraction to

[batch_size×num_modalities,channels,height,width].

This treats all modality images across all samples as a single large batch, allowing the feature extractor to process them efficiently. After extraction, the output tensor is reshaped back to [batch_size,num_modalities,feature_dim] and finally flattened to [batch_size,num_modalities×feature_dim] for dimensionality reduction and classification.

This procedure implements feature-level concatenation of the seven wrist-derived modalities. It does not define an additional training strategy or a separate fusion model; instead, it is the computational implementation used to obtain the fused representation for each 30 s window. Due to the effective batch size of [batch_size×num_modalities], we used a smaller batch size of 16 during training. For example, with seven sensor modalities, this results in 112 images being processed per forward pass.

### 6.4. Dimensionality Reduction

The fusion step produces high-dimensional feature vectors by concatenating features extracted from each sensor modality. To reduce the computational burden and potentially improve generalization, we introduce a dimensionality reduction step using **PCA**.

PCA was implemented using the scikit-learn library and applied in two stages:

1. **PCA Fit (Training Phase)**: As PCA is an unsupervised algorithm, it does not require class labels. To learn the transformation matrix, PCA was fitted using an auxiliary set of unlabeled signal-encoded samples. These samples came from recorded scenarios or intervals that contained valid Empatica E4 physiological and motion signals but were not assigned to any of the four operational classes. Therefore, they were not labeled as LL, L, H, or HH; were not used as supervised classifier targets; and were not included in the supervised evaluation folds as labeled test samples. The user composition of the auxiliary unlabeled PCA set followed the corresponding modeling configuration. In the target-driver-only configuration, PCA was fitted using unlabeled samples from the target driver. In the external-user-only configuration, PCA was fitted using unlabeled samples from the external users. In the mixed target/external-user configuration, PCA was fitted using unlabeled samples from both the target driver and the external users. Thus, when external-user data were incorporated into a modeling configuration, they were incorporated consistently both in the unsupervised PCA fitting stage and in the supervised classifier training stage, while the PCA stage itself remained label-free. Each image was first processed by the frozen ResNet-50 feature extractor, and the resulting feature vectors were collected across batches. A batch size of 256 was used during this phase to ensure efficient processing. After collecting all feature vectors, they were concatenated into a single matrix, which served as input for fitting the PCA model. The number of principal components was set to 100. In the LOEO evaluation, this PCA fitting procedure was repeated independently for each fold, using only training-side data from the corresponding modeling configuration and excluding all labeled and unlabeled windows from the held-out target-driver experience.
2. **PCA Transform (Application Phase)**: Once fitted, the PCA transformation was used to project high-dimensional feature vectors onto a lower-dimensional subspace before classification. This PCA step should be interpreted as a configuration-specific unsupervised representation calibration step. It estimates a compact feature space from auxiliary unlabeled recordings associated with the corresponding modeling configuration, without using LL, L, H, or HH labels and without optimizing the supervised classifier. The supervised classifier was trained only with labeled samples from the corresponding training configuration and evaluated on the labeled target-driver test data. No image from the held-out LOEO test experience was used to fit PCA, train the supervised classifier, or select the final model.

The reduction in dimensionality depends on the choice of feature extractor. ResNet-50 produces a 14,336-dimensional vector (2048 features × 7 modalities), which is compressed to 100 components, yielding a compression ratio of approximately 99.3%.

This PCA-based step significantly reduces the input size for the final classifier, allowing for faster training and inference while retaining the most informative features.

### 6.5. Classification

The input to the classifier consists of a 100-dimensional vector obtained from PCA. The classifier architecture is defined as follows:

- **Input**: 100-dimensional vector from PCA.
- **Fully Connected Layer 1**: Reduces the input from 100 to 25 neurons.
- **Activation**: Rectified Linear Unit (ReLU).
- **Fully Connected Layer 2**: Reduces from 25 to 10 neurons.
- **Activation**: ReLU.
- **Fully Connected Layer 3 (Output Layer)**: Maps from 10 neurons to 4 output classes corresponding to LL, L, H, and HH.

#### Training and Evaluation Configuration

Only the final classifier was trained in a supervised manner; the ResNet-50 feature extractor remained frozen. Two evaluation protocols were used, with different roles in the manuscript:

1. **Leave-One-Experience-Out (LOEO):** This is the primary evaluation protocol. For User 1, the model is trained on all valid target-driver experiences except one and evaluated on the held-out experience. In each LOEO fold, the held-out experience was used exclusively as the test set, while the windows from the remaining non-held-out experiences were split into training and validation subsets using an 85%/15% partition. The validation subset was used for model selection based on validation loss, and the reported metrics were computed only on the held-out test experience. Experiences 4 and 5 were excluded from the reported LOEO fold set because their labeled-window distributions were especially skewed toward the L class, corresponding to manual driving. This imbalance was particularly notable in these two sessions: real-world timing constraints produced manual-driving intervals that were substantially longer than expected. Because no class rebalancing, class-weighted loss, or resampling strategy was applied, their use as held-out folds would provide limited evidence about discrimination among H, HH, and the remaining operational classes. The reported mean LOEO metrics therefore characterize session-level generalization across the seven retained experiences and do not quantify generalization to Experiences 4 and 5. They should not be interpreted as an average over all nine valid target-driver sessions. This protocol tests generalization to unseen driving sessions and reduces the risk of information leakage caused by temporally overlapping sliding windows. User 1 was treated as the primary target driver because this participant provided the largest number of valid repeated sessions, making a session-level LOEO evaluation methodologically interpretable. Users 2, 3, and 4 were not used as equivalent target drivers because they provided only 4, 3, and 2 valid sessions, respectively. With so few independent sessions, LOEO estimates would be dominated by fold-specific class distributions and session idiosyncrasies rather than by stable predictive generalization.
2. **Random Sampling (RS):** This protocol randomly splits the available data into training (70%), validation (15%), and test (15%) partitions. As in LOEO, validation data were used for model selection based on validation loss, and the reported metrics were computed only on the test partition. Because adjacent windows overlap, RS is treated as a development-oriented protocol for ablation analyses and methodological comparisons, not as the main evidence of session-level generalization.

The training configuration for all experiments is as follows: batch size of 16, number of epochs set to 25, and early model selection based on the lowest validation error. The loss function used is CrossEntropyLoss, which internally applies the softmax activation to the output layer. The optimizer is Adam with a learning rate of 0.001 and weight decay of $1\times 10^{-4}$; all other Adam parameters were kept at their PyTorch default values. Default weight initialization was used. No oversampling, undersampling, synthetic sample generation, class-weighted loss, or sample-weighted loss was applied during supervised classifier training.

Training was conducted using PyTorch 2.1 with CUDA 12.1 on a system equipped with an NVIDIA RTX A4000 GPU (16 GB), an Intel Core i9 CPU, and 64 GB of RAM. For full implementation details and reproducibility instructions, please refer to the accompanying GitHub repository.

### 6.6. Evaluation Protocols and Metrics

In DMSs, the practical implications of different error types can vary. For instance, incorrectly categorizing higher-demand operational classes (H/HH) as lower-demand classes (LL/L) may result in a delayed response, while over-detecting high demand may lead to the triggering of unnecessary alerts or interventions. For this reason, we report multiple metrics beyond accuracy to better characterize performance under class imbalance.

The evaluation metrics defined in Equations (9)–(13) are selected to provide a comprehensive and balanced assessment of model performance in our **multi-class, imbalanced classification** setting.

Equation (9) defines the overall **accuracy (A)**, measuring the proportion of correctly classified samples. While widely used, accuracy can be misleading in imbalanced datasets, as it tends to reflect performance on the majority classes more strongly than on the minority ones.

To address this, we report **macro-averaged precision (P), recall (R), and F1-score (F1)**, given in Equations (10), (11), and (12), respectively. These metrics are computed by first evaluating each metric per class and then averaging over all *C* classes. This macro-averaging approach assigns equal importance to each class, regardless of its prevalence in the dataset, providing a more informative measure of overall classifier performance in the presence of class imbalance [69]. The same family of metrics is also commonly reported in recent driver-state and physiological-signal classification studies, supporting their use as complementary criteria for evaluating classification performance [70,71].

Additionally, we include **Cohen’s kappa coefficient (κ)**, defined in Equation (13), which quantifies the agreement between predicted and true labels while correcting for agreement expected by chance. Unlike raw accuracy, κ accounts for chance agreement under the observed marginal distributions of the confusion matrix, which makes it informative in skewed classification settings [72]. Interpretation of κ follows the widely adopted scale by Landis and Koch [73]:

- κ≤0: Poor agreement,
- 0.01–0.20: Slight agreement,
- 0.21–0.40: Fair agreement,
- 0.41–0.60: Moderate agreement,
- 0.61–0.80: Substantial agreement,
- 0.81–1.00: Almost perfect agreement.

Together, these metrics form a comprehensive evaluation suite: *macro-averaging* ensures that the classifier’s performance is not dominated by common classes, while *Cohen’s kappa* quantifies the reliability of classification beyond chance.

(9) $$\mathrm{Accuracy}\;(\%)=\;\frac{1}{N}\sum_{i=1}^{N}1\left[{\hat{y}}_{i}\;=\;y_{i}\right]\cdot 100$$

(10) $$\mathrm{Precision}\;\left(\%\right)\;=\;\frac{1}{C}\sum_{i=1}^{C}\frac{{\mathrm{TP}}_{i}}{{\mathrm{TP}}_{i}+{\mathrm{FP}}_{i}}\cdot 100$$

(11) $$\mathrm{Recall}\;\left(\%\right)\;=\;\frac{1}{C}\sum_{i=1}^{C}\frac{{\mathrm{TP}}_{i}}{{\mathrm{TP}}_{i}+{\mathrm{FN}}_{i}}\cdot 100$$

(12) $$F_{1}\text{-}\mathrm{Score}\;\left(\%\right)\;=\;\frac{1}{C}\sum_{i=1}^{C}2\cdot \frac{{\mathrm{Precision}}_{i}\cdot {\mathrm{Recall}}_{i}}{{\mathrm{Precision}}_{i}+{\mathrm{Recall}}_{i}}\cdot 100$$

(13) $$\kappa \;=\;\frac{p_{o}-p_{e}}{1-p_{e}}$$

where:

- *C* is the number of classes;
- *N* is the total number of samples;
- ${\hat{y}}_{i}$ is the predicted label for sample *i*;
- $y_{i}$ is the true label for sample *i*;
- ${\mathrm{TP}}_{i}$, ${\mathrm{FP}}_{i}$, and ${\mathrm{FN}}_{i}$ refer to the true positives, false positives, and false negatives for class *i*;
- $p_{o}$ is the observed agreement (accuracy);
- $p_{e}$ is the expected agreement by chance, calculated from the confusion matrix.

## 7. Results

This section reports the experimental results obtained with the wearable-signal modeling pipeline described in Section 6. The experiments use continuous RP for signal-to-image encoding, a frozen ResNet-50 feature extractor, PCA-based dimensionality reduction to 100 components, and the classifier architecture described above. Unless otherwise stated, the primary evidence corresponds to the LOEO target-driver evaluation for User 1. The reported results should therefore be interpreted as a target-driver feasibility evaluation under the available real-world data, not as population-level evidence of general superiority of personalized models.

### 7.1. Intra-Subject Approach

We evaluated the model using the LOEO strategy on data from User 1, who was treated as the primary target driver. Three mutually exclusive training configurations were compared within this target-driver setting: (1) training on data from other users only, (2) training on a combination of data from other users and the target user, and (3) training solely on data from the target user. For each configuration, the model was trained from scratch for every fold.

**Figure 5:**
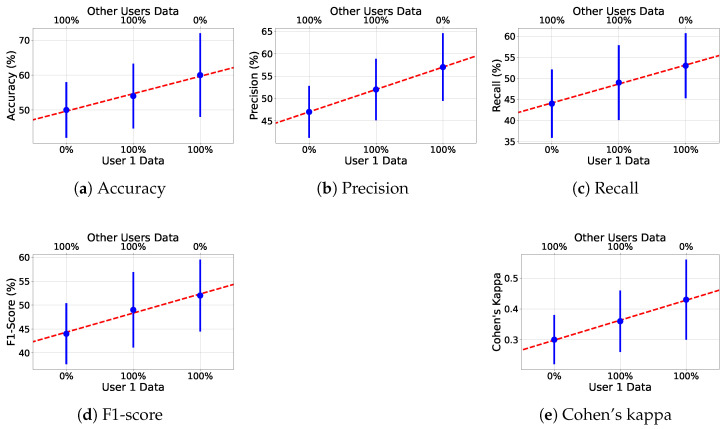
LOEO evaluation results across five performance metrics: accuracy, precision, recall, F1-score, and Cohen’s kappa.

Figure 5 presents the mean performance across the seven LOEO folds for five evaluation metrics. In all cases, performance improved as training data became more tailored to the target user. For User 1, accuracy increased from 50% when trained on other users only to 54% with combined user data and reached 60% when trained solely on the target user. Precision improved from 47% to 52% and 57%, recall from 44% to 49% and 53%, and F1-score from 44% to 49% and 52%. Similarly, Cohen’s kappa increased from 0.30 to 0.36 and 0.43, respectively.

These results suggest a consistent target-driver pattern: target-driver-only training improved the classification of the scenario-derived operational driver-state classes across all evaluated metrics. This finding is consistent with Hypothesis 1, since wrist-derived physiological and motion signals provided discriminative information for User 1 under real-world SAE Level 2 conditions. In addition, the lower performance obtained when the available external-user data were used, either alone or combined with target-driver data, is consistent with Hypothesis 2 in the evaluated primary target-driver setting. Overall, this pattern provides initial feasibility evidence for personalized wearable-signal modeling of the operational classes under the evaluated real-world conditions.

### 7.2. Detailed Performance Analysis

**Figure 6:**
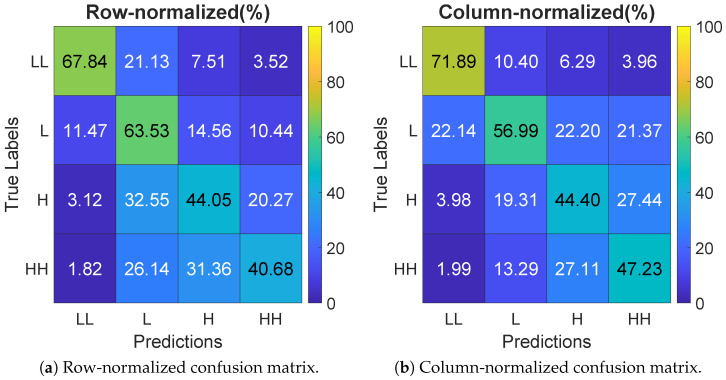
Confusion matrices obtained from the LOEO evaluation. The row-normalized matrix shows class-wise recall, while the column-normalized matrix shows class-wise precision.

A detailed evaluation of classifier performance was conducted using the LOEO cross-validation strategy for the target-driver-only configuration. To obtain aggregated performance metrics, confusion matrices were computed for each fold and then combined. Figure 6a presents the row-normalized confusion matrix, highlighting the classifier’s recall (true positive rate) for each class, while Figure 6b shows the column-normalized version, which reflects the classifier’s precision across classes.

The global performance metrics, computed over all LOEO folds, are as follows: overall accuracy (A) = 60%, macro-averaged precision (P) = 57%, recall (R) = 53%, F1-score = 52%, and Cohen’s kappa (κ) = 0.43. These values indicate moderate overall classification performance and moderate agreement beyond chance.

Figure 6a provides the recall-oriented view of the classifier. In this matrix, each row corresponds to the distribution of predictions for one true class. The diagonal values show that LL and L are the most consistently recovered classes, with 67.84% and 63.53% of their true windows correctly assigned, respectively. H and HH show lower recall, with 44.05% and 40.68% correct assignments. Most H errors are assigned to L (32.55%) or HH (20.27%), while most HH errors are assigned to H (31.36%) or L (26.14%). This pattern indicates that the model separates LL and L more clearly than H and HH.

Figure 6b provides the precision-oriented view. In this matrix, each column describes the true-class composition of the samples assigned to a predicted class. Predictions of LL are the most reliable, with 71.89% of samples predicted as LL actually belonging to LL. Predictions of H and HH are less precise, with 44.40% and 47.23%, respectively, because these predicted categories also include samples from related operational classes. This behavior is consistent with the protocol structure: H and HH are closer operationally than LL, which is reflected in their higher mutual confusion.

However, performance is not evenly distributed across the four scenario-derived operational driver-state classes, as illustrated in Figure 6. This class-wise imbalance is further clarified in Table 6, which presents per-class metrics under a one-vs.-rest scheme. The classifier performs best on the LL class, achieving the highest accuracy (88.4%), precision (72%), recall (68%), F1-score (70%), and Cohen’s kappa (0.52). In contrast, performance on the H and HH classes is lower, with F1-scores of 44% and Cohen’s kappa values around 0.27–0.30, indicating confusion between the higher-demand operational classes. For DMS research, the LL class appears to be the easiest to detect in our setting, suggesting that the pipeline can distinguish parked low-demand baseline intervals from more active driving and DAS-engaged operational intervals.

It is important to note that the per-class accuracy values are relatively high compared to F1-scores. This discrepancy arises from the class imbalance inherent in multi-class metrics: for a given class treated as positive, all samples from the remaining classes are counted as true negatives, which artificially inflates the accuracy. Therefore, metrics such as precision, recall, and F1-score provide a more informative assessment of classifier effectiveness in this context. The normalized confusion matrices also indicate that the reported performance is not explained by a trivial collapse toward a single majority class. The row-normalized matrix shows that the classifier recovered 67.84% of LL windows and 63.53% of L windows, while H and HH were also recovered with recalls of 44.05% and 40.68%, respectively. The main limitation is therefore not a simple majority-class prediction pattern but the weaker separation between the higher-demand operational classes, especially H and HH.

### 7.3. Ablation Studies

The following ablations are interpreted as methodological analyses.

#### 7.3.1. Impact of PCA Dimensionality on Model Convergence

To reduce the dimensionality of the feature space prior to classification, PCA was applied. We evaluated three configurations with 50, 100, and 200 components, resulting in different input sizes for the classifier. The network architecture remained constant across experiments: the first dense layer projected the PCA-reduced input to 25 neurons, followed by a second layer with 10 neurons and a final output layer predicting one of the four scenario-derived operational driver-state classes.

**Figure 7:**
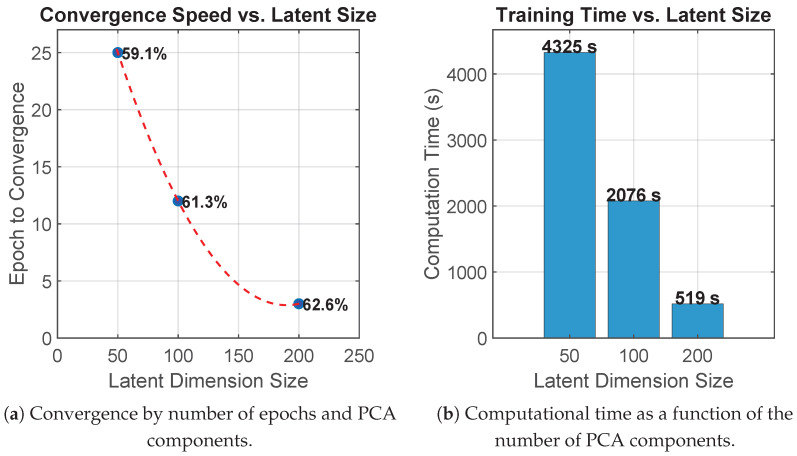
Effect of PCA dimensionality on classifier convergence and training efficiency.

Figure 7 summarizes the impact of PCA dimensionality on classification performance and training dynamics. As shown in Figure 7a, higher-dimensional inputs yielded marginal improvements in accuracy: 59.1% with 50 components, 61.3% with 100, and 62.6% with 200, an overall gain of 3.5 percentage points. More notably, the number of epochs required to reach peak performance decreased substantially: 25 epochs for 50 components, 13 for 100, and only 3 for 200, indicating faster convergence. This trend may also signal increased risk of overfitting at higher dimensions.

Training time per epoch was consistent across configurations, averaging approximately 173 s using the LOEO dataset division. However, due to differences in number of epochs to convergence, total training durations varied significantly: 4325 s for the 50-component model, 2076 s for the 100-component model, and just 519 s for the 200-component configuration (Figure 7b). While higher-dimensional inputs enabled faster convergence, the potential for overfitting necessitates a trade-off between accuracy, training efficiency, and generalizability in real-world scenarios.

#### 7.3.2. Evaluating the Role of Wrist-Derived Signals in Personalized Model Performance

Are all wrist-derived streams equally informative for model performance? To investigate this question, we performed additional experiments using the RS protocol, evaluating models under different input configurations. We maintained the same training setup as in prior analyses, varying only the number and combination of input signals. Eleven configurations were tested and performance was assessed using overall accuracy and Cohen’s kappa. The complete results are presented in Table 7.

In this analysis, the physiology-only configuration refers to the Empatica E4 streams BVP, EDA, HR, and TEMP. Wrist acceleration was treated separately as a motion-related input and included the three accelerometer axes ACCEL_X, ACCEL_Y, and ACCEL_Z. Therefore, the comparison distinguishes between physiology-only information, acceleration-only information, and their feature-level fusion.

The relatively strong performance of the acceleration-only configuration indicates that the classification task may partly capture motion and posture patterns induced by the protocol, not only autonomic physiological responses. This interpretation is coherent with the scenario-derived nature of the labels, since several operational classes involve scripted head, hand, or maneuver-related actions. Therefore, the fused model should be understood as a wearable physiological-and-motion classifier, rather than as a purely autonomic physiological-state estimator.

The results indicate that not all streams contribute equally to classification performance. For instance, training with acceleration data alone yielded an accuracy of 52.9% and a Cohen’s kappa of 0.388, indicating moderate agreement and suggesting that motion-related information contributes substantially to the classification. This outperformed classifiers trained on individual physiological streams such as BVP, HR, EDA, or temperature, all of which resulted in kappa values below 0.13, suggesting only slight or negligible agreement. Temperature alone produced a kappa near zero, performing similarly to a random classifier.

Notably, the physiology-only configuration, which combined BVP, EDA, HR, and TEMP while excluding wrist acceleration, achieved 58.2% accuracy and a kappa of 0.483, exceeding the performance of the acceleration-only model. This suggests that autonomic wearable streams still provide complementary information, even though none of them was highly discriminative when used individually. Moreover, excluding HR from the full wearable-signal configuration unexpectedly boosted performance, achieving the best overall results, with 65.8% accuracy and a kappa of 0.525. This suggests that in this context, HR may introduce noise or redundancy that impairs model generalization.

#### 7.3.3. Comparison of Techniques for Transforming Signals into Images

To select the most effective technique for transforming one-dimensional time series signals into two-dimensional image representations, we conducted a comparative evaluation of established signal-to-image encoding methods. As shown in Table 8, continuous RP achieved the highest classification accuracy (64.2%), outperforming all other techniques, including binary RP, GASF, GADF, and MTF at both low (4 states) and high (128 states) discretization levels. These results support the adoption of continuous RP as the preferred signal-to-image encoding method within the evaluated pipeline.

#### 7.3.4. Division of the Dataset

To facilitate efficient experimentation during model development, we used a random sampling strategy as a development-oriented protocol. This approach reduced computational cost because each methodological comparison could be evaluated without retraining the model once per held-out session. However, because the dataset was generated using 30 s windows with a 3 s step, adjacent samples may overlap substantially. Therefore, RS can be affected by temporal leakage if overlapping windows from the same driving interval are split between training and test partitions. For this reason, RS is not treated as the primary evidence of session-level generalization.

We compared RS with LOEO to assess whether the selected windowing configuration produced clearly inflated estimates. LOEO is the stricter protocol because it holds out complete driving experiences, thereby reducing the risk that temporally adjacent windows from the same session appear in both training and test sets. Accordingly, LOEO is used as the main evaluation protocol, while RS is used to support faster exploratory analyses.

For this comparison, we used the dataset from User 1. RS was repeated seven times, matching the number of LOEO folds, and the results were aggregated across repetitions.

As shown in Table 9, RS and LOEO produced mean performance values of the same order of magnitude in this specific configuration. RS achieved an accuracy of **60.20% ± 6.06%** and a Cohen’s kappa of **0.486 ± 0.11**, while LOEO achieved **59.32% ± 12.98%** accuracy and **0.4307 ± 0.15** kappa. This comparison should not be interpreted as proving that RS and LOEO are equivalent. LOEO remains the primary evidence of session-level generalization. The RS results are reported only as development-oriented analyses, and their interpretation is limited by the temporal overlap between adjacent sliding windows.

The lower standard deviation obtained with RS is expected because random partitions mix windows from the available sessions and therefore reduce session-to-session variability. In contrast, the higher LOEO variability reflects the real difficulty of generalizing across distinct driving experiences from the same target driver. This variability is relevant for personalized DMSs, where repeated sessions from the same user may still differ because of traffic, context, sensor placement, and physiological state drift.

Additional tests with different window lengths confirmed the sensitivity of RS to temporal overlap. A larger 120 s window produced inflated performance of approximately 90%, consistent with leakage effects, whereas a shorter 10 s window reduced accuracy below 50%. Taken together, these results support the use of RS as a practical tool for early methodological development, provided that windowing choices are checked against a session-level protocol such as LOEO before drawing conclusions.

### 7.4. Computational Performance and Offline Inference Latency

An offline inference benchmark was conducted to characterize the computational cost of the proposed pipeline on the workstation described in Section Training and Evaluation Configuration. The benchmark used previously recorded wrist-derived physiological and motion signals loaded into memory before measurement. Each iteration started from one complete 30 s multimodal window and processed it using a batch size of one. The measured scope therefore comprises the processing stages applied after signal acquisition.

The evaluated pipeline comprised the conversion of the seven wrist-derived streams into continuous Recurrence Plot images, feature extraction using the frozen ResNet-50 model in evaluation mode, feature-level fusion, PCA projection, and classification using the trained MLP. Sensor acquisition, communication, cross-device synchronization, disk input/output, and deployment-specific scheduling fall outside the measured scope.

Before measurement, 1,000 warm-up inferences were executed and excluded from the analysis. The complete pipeline was then executed continuously for one hour, producing 239,413 measured inferences. Gradient computation was disabled, and CUDA synchronization was applied during timing to account for asynchronous GPU execution. Latency was recorded separately for each processing stage and for the complete pipeline. The mean and the P50, P90, P95, and P99 percentiles were calculated from the raw latency measurements.

As shown in Table 10, the measured offline processing pipeline achieved a mean latency of 15.020 ms per previously acquired 30 s multimodal window. The P95 and P99 latencies were 18.105 ms and 19.129 ms, respectively, corresponding to an average observed processing rate of approximately 66.5 windows per second.

ResNet-50 feature extraction accounted for the largest share of the measured latency, followed by signal-to-image conversion and PCA projection. Feature fusion and MLP classification required comparatively little processing time. On the evaluated workstation, the measured processing latency was substantially shorter than the 3 s step used between successive windows. This comparison refers exclusively to the offline processing stages included in the benchmark.

## 8. Discussion

### 8.1. Real-World Experimentation

Simulator studies provide repeatability and experimental control for investigating human–automation interaction and psychophysiological responses [9,10,11,74]. By contrast, real-world datasets capture naturalistic driver and biometric variability under operational conditions [75]. The present study therefore used on-road experiments to obtain more contextually grounded responses. Naturalistic evidence from commercial trucking suggests that ADAS deployment can measurably alter driver behavior and performance [76]. This supports the need for on-road experimentation, where behavioral adaptation, physiological variability, and real-world uncertainty are present and can shape the recorded signals. While simulations offer controllability and safety, they may lack the ecological validity necessary to capture authentic behavioral and physiological responses. In contrast, driving in the real world inherently involves dynamic uncertainty and genuine risk, including the potential for accidents, which increases the relevance of evaluating driver-monitoring pipelines under operational conditions [1].

The present study does not treat real-world experimentation as a substitute for simulator-based research but as a necessary complementary step for evaluating DMS pipelines under operational constraints. Deploying experiments in operational vehicles introduces additional complexities, including restrictions on driver eligibility, sensor integration within the vehicle cabin, synchronization across independent devices, and the unpredictability of traffic and environmental conditions. These constraints directly affected the experimental protocol. For example, a lane change scenario could not always be executed when traffic density was high, when the adjacent lane was occupied, or when the co-driver considered that the maneuver should be delayed for safety reasons. In these cases, the scenario was interrupted, shortened, or excluded from the valid labeled intervals. Because supervised modeling required reliable intervals for the complete set of operational classes, such interruptions could invalidate an entire 30–45 min session for dataset construction. Because the scenarios followed a fixed sequence, possible temporal effects such as habituation, anticipation, or fatigue cannot be completely ruled out. However, the objective of the protocol was to obtain reproducible scenario-derived operational intervals under safe public-road conditions, and future protocols may consider counterbalancing or constrained randomization when compatible with on-road safety requirements.

This illustrates one of the methodological lessons of the work: conditions that are controllable in simulation become part of the experimental problem in public-road testing, where the experimental vehicle interacts with other vehicles and where safety supervision takes priority over strict scenario timing. Despite these challenges, the advantages of real-world experimentation, especially for assessing driver-state sensing in safety-relevant scenarios, justify its adoption in this study and position it as a valuable step for advancing driver monitoring systems beyond controlled environments.

### 8.2. Sensor Selection

Sensor selection is a key consideration in developing DMSs, particularly when moving from controlled environments to real-world driving. High-precision modalities such as ECG and EEG remain highly valuable for studying autonomic and central nervous system responses, and recent wearable, dry-electrode, capacitive, and in-vehicle implementations are progressively reducing their intrusiveness [46,47]. These modalities are especially useful for controlled experiments, validation-oriented studies, and protocols designed to characterize specific physiological mechanisms. Physiological states of vehicle occupants have also been incorporated into autonomous-vehicle safety decision-making using near-infrared spectroscopy [77]. For the present design, however, the focus was placed on a lower-intrusion configuration that could be applied repeatedly during on-road sessions with minimal setup burden. Findings from these modalities are therefore interpreted as complementary evidence, while the evaluated pipeline focuses on wrist-derived physiological and motion signals.

Remote physiological sensing may ultimately become a highly effective solution for in-cabin driver monitoring, as camera- or radar-based methods can estimate vital signs without requiring the driver to wear a device. Recent rPPG studies and in-vehicle datasets demonstrate significant advancements in this area; however, they also highlight ongoing challenges related to dynamic lighting, head motion, vehicle vibration, limited dataset diversity, and cross-dataset generalization [49,50]. As these methods continue to evolve, they may become a more user-friendly alternative to wristbands, although privacy, robustness, and cross-dataset generalization remain important deployment challenges. In the present work, wrist-worn sensing offers a practical intermediate solution: it is less intrusive than electrode-based acquisition, currently easier to deploy repeatedly than remote physiological estimation in unconstrained driving, and does not interfere with vehicle safety systems such as seat belts or airbags.

Wearable devices like wristbands offer a less intrusive and more scalable alternative. Although they typically have lower sampling rates and reduced signal fidelity, they enable continuous monitoring without interfering with driving behavior or ergonomics. In this study, we selected the Empatica E4 wristband for its ease of integration, built-in preprocessing, and proven performance in affective computing applications [78]. While less precise than clinical-grade sensors, its design meets the practical demands of real-world DMS [16]. Fully remote physiological sensing technologies remain insufficiently mature for robust deployment in dynamic driving conditions [79], making wrist-mounted sensing a pragmatic compromise between usability and signal quality.

This choice should not be interpreted as a claim that wristbands are the only valid non-intrusive sensing modality. Rather, the Empatica E4 was selected as a compromise between intrusive physiological sensing and fully remote sensing approaches. This compromise is consistent with the broader literature, which supports multimodal and wearable sensing for driver monitoring while acknowledging the practical constraints of real-world deployment [17,18]. The acquisition platform was multimodal, but the classifier evaluated in this manuscript focuses on the wrist-derived BVP, EDA, HR, skin temperature, and acceleration streams, in direct alignment with Hypothesis 1. Thus, the reported results should be interpreted as evidence that non-intrusive wearable physiological and motion signals can support the proposed target-driver classification task.

### 8.3. Operational Driver-State Label Nomenclature

Interpreting internal driver states from physiological signals is challenging, especially in real-world driving, where physiological responses may be affected by motion, traffic context, sensor placement, and individual variability. Prior work has shown that physiological signals can be informative for studying awareness-related transitions and internal human states [80,81]. However, such constructs require careful experimental definition and validation. For this reason, the present work does not treat the proposed labels as direct measurements of awareness, workload, stress, distraction, drowsiness, trust, or situation awareness.

In this study, LL, L, H, and HH are defined as *scenario-derived operational driver-state classes*, organizing predefined experimental phases and scenario groups into four ordered supervised learning categories. The labels are directly tied to the experimental protocol: parked baseline, manual driving, DAS-engaged scenarios with scripted gaze or head-orientation conditions, and DAS-engaged scenarios involving lane changes or sudden acoustic startle events. This terminology is used to ensure that the classification task is based on available ground truth derived from protocol timing, scenario execution records, and offline visual verification.

This distinction matters because established constructs such as workload, attention, distraction, stress, trust, and situation awareness are normally assessed using validated protocols, psychometric instruments, behavioral measures, or dedicated experimental manipulations. Workload, for instance, is commonly assessed using instruments such as the NASA Task Load Index (NASA-TLX) [82], while situation awareness has a well-developed theoretical and measurement framework of its own [83]. Other studies have employed physiological signals such as EEG or Galvanic Skin Response (GSR) to model constructs related to trust, situation awareness, or engagement [84,85,86,87]. The present study is situated within this broader line of work, but it does not adopt these constructs as validated ground-truth labels.

Expert visual review was therefore used to confirm that each protocol interval was correctly executed and that the corresponding sensor data were valid, not to infer latent psychological states independently of the scenario structure. The resulting labels should accordingly be interpreted as operational proxies for experimentally defined driving conditions, not as validated psychological or psychophysiological constructs.

### 8.4. Physiological and Motion-Related Contributions

The ablation results clarify the type of information captured by the proposed wearable-signal classifier. In the RS development-oriented analysis, the acceleration-only configuration achieved 52.9% accuracy and a Cohen’s kappa of 0.388, whereas the physiology-only configuration based on BVP, EDA, HR, and TEMP achieved 58.2% accuracy and a kappa of 0.483. The full wearable-signal configuration reached 64.2% accuracy and a kappa of 0.506, and the best configuration, excluding HR, reached 65.8% accuracy and a kappa of 0.525. These results suggest that wrist acceleration captures relevant protocol-induced motor and postural patterns, while the physiological streams provide complementary information that is not fully explained by motion alone. This interpretation is consistent with the scenario-derived nature of the labels, since several classes include observable actions or operational events, such as head orientation, hand position, lane changes, and sudden acoustic startle events. Previous work on smartwatch-based driver monitoring has also shown that wearable motion and contextual signals can support the classification of driver activities and driving events in naturalistic conditions [17].

These findings support a practical interpretation of the proposed system as a wearable physiological-and-motion classifier for scenario-derived operational classes. Motion-related information can help identify protocol-relevant behavior, and the stronger performance of the physiology-only configuration compared with acceleration alone indicates that autonomic wearable streams also contribute to the task. At the same time, wrist-worn physiological signals can be affected by motion artifacts and sensor-contact variability, particularly in BVP and EDA recordings [88]. Future work should therefore investigate more specific physiological preprocessing, motion-artifact correction, and multimodal fusion with camera-based behavioral features to separate autonomic responses and motor patterns more clearly.

The confusion between H and HH observed in both normalized confusion matrices is consistent with a combination of protocol, sensing, and data-related factors. Both classes are derived from DAS-engaged scenarios, and their operational distinction is finer than the separation between the parked baseline, manual driving, and DAS-engaged conditions. H groups scripted head-orientation and dashboard-gaze scenarios, whereas HH combines lane change and sudden acoustic startle events; several scenarios across these groups also share related gaze and hand-position instructions. Since the wearable is located at the wrist, the resulting wrist-motion patterns may be similar across some of these conditions, particularly when the distinctive event occupies only part of the 30 s analysis window. Physiological responses may also overlap between H and HH and vary in magnitude and timing across repetitions, reducing consistent separation in BVP, EDA, HR, and skin temperature. The available number of independent target-driver sessions may further limit the diversity of H and HH patterns represented during training. The observed confusion is therefore interpreted as the combined effect of the operational proximity of the scenarios, similarity in wrist-derived motion patterns, temporal aggregation, within-driver physiological variability, and the available sample diversity. Scenario-level and event-aligned analyses would allow the contribution of these factors to be examined separately in future work.

### 8.5. Classification Model

The classification model used a ResNet-50 backbone pre-trained on ImageNet as a fixed feature extractor [89,90]. This choice was motivated by the limited amount of labeled target-driver data and the need to obtain a compact image representation without training a large convolutional model from scratch. In this pipeline, each signal-encoded image is represented by the 2,048-dimensional output of the global average pooling layer. Other feature extractors could also be justified within the same architecture, including alternative CNN backbones, self-supervised encoders, autoencoders, or task-specific neural feature extractors. Recent driver-state recognition studies further support the use of deep architectures in this research area [4].

PCA was then applied as a simple and efficient dimensionality-reduction step, reducing the fused 14,336-dimensional representation to 100 components before supervised classification. We are aware that PCA applies a linear transformation between the input feature space and the reduced representation. In the proposed pipeline, this linear reduction is placed between two nonlinear stages: the ResNet-50 feature extractor before PCA and the ReLU-based classifier after PCA. Nonlinear alternatives could also be explored in future work. Kernel PCA [91] and Uniform Manifold Approximation and Projection (UMAP) could be evaluated as alternative dimensionality reduction methods [92], while t-distributed Stochastic Neighbor Embedding (t-SNE) could be useful for visual inspection of the learned feature space [93].

This design also reflects a practical constraint of the dataset. Reliable operational labels are time-consuming to assign in real-world driving because scenario execution, synchronization, and signal availability must be checked carefully [66,94]. In this study, more than 152,000 unlabeled image samples were available for auxiliary PCA fitting, whereas the supervised dataset contained 5025 labeled samples overall, including 2158 samples from the primary target driver.

Although the primary LOEO evaluation was conducted on a single target participant (User 1), data from three additional users (Users 2, 3, and 4) were also used to evaluate whether the available external-user information improved performance in the same target-driver setting. The results showed that for the evaluated target driver, integrating the available external-user data did not enhance performance; instead, it led to lower scores across the evaluated metrics. This pattern is consistent with Hypothesis 2, which states that in an intensive longitudinal target-driver setting, intra-subject training provides better session-level classification performance for the target driver than configurations incorporating external-user data.

The results are also consistent with Hypothesis 1, but for a different reason: the target-driver-only LOEO evaluation shows that non-intrusive wrist-derived physiological and motion signals contain discriminative information for the proposed scenario-derived operational driver-state classes under real-world SAE Level 2 automated driving. Consequently, our methodology prioritizes repeated observations from the target driver, since personalized driver-state sensing depends on capturing user-specific physiological and motion patterns across sessions. This does not imply that larger cohorts are unnecessary; rather, the present work evaluates an initial target-driver feasibility step. This strategy is consistent with recent advances in human–machine interaction, which highlight the importance of modeling user-specific baselines and responses rather than relying only on generalized population-level patterns [95].

To transform temporal physiological signals into a format compatible with vision-based deep learning, we evaluated three well-established time-series-to-image encoding methods: RP, GAF, and MTF. These techniques are widely recognized for their ability to capture temporal dynamics as spatial patterns and have been previously applied in affective computing contexts involving physiological signals [52,96,97]. While numerous alternative transformations exist—such as spectrograms, scalograms derived from wavelet transforms, visibility graphs, and Hilbert space-filling curves—a comprehensive benchmarking of all available methods lies beyond the scope of this study [98]. Instead, our objective was to establish a robust and interpretable baseline tailored to the classification of physiological signals. The experimental results suggest that continuous-RP offers a favorable balance between visual expressiveness and classification performance within the specified framework. The evaluated signal-to-image encodings capture temporal structure within each 30 s window. Longer-term dependencies across successive windows and complete driving sessions fall outside the present modeling scope. Many studies in the literature (e.g., [88,99]) include dedicated preprocessing of physiological signals, such as artifact removal, filtering, and handcrafted feature extraction. In this work, preprocessing was limited to uniform resampling so that the image-based representation pipeline could operate on consistently processed input streams. This choice establishes a simple and reproducible baseline before introducing more specialized physiological preprocessing in future work.

### 8.6. Dataset Partitioning Strategy

This work reports results using both LOEO and RS strategies for dataset partitioning. As shown in the Section 7, RS produced performance metrics of the same order of magnitude as LOEO in the selected configuration while reducing computational cost, since only a single training run is required instead of *n* separate runs, where *n* corresponds to the number of sessions. However, caution is warranted when interpreting such results. As discussed in the literature, RS can lead to overly optimistic performance estimates due to information leakage between training and test sets, particularly in time-dependent or user-specific data such as human activity or driver-state recognition [100].

For this reason, LOEO is treated as the primary evaluation protocol in this manuscript. LOEO holds out complete driving experiences and is therefore more appropriate for assessing session-level generalization in the presence of overlapping sliding windows. RS is retained only as a development-oriented analysis, not as the main evidence of generalization. In our experiments, RS and LOEO produced mean accuracy values of the same order of magnitude under the selected 30 s window and 3 s step, but this should not be interpreted as proof that both protocols are equivalent. The higher variability observed in LOEO reflects the difficulty of generalizing across distinct real-world driving sessions from the same target driver.

Experiences 4 and 5 were valid recorded sessions that satisfied the dataset-generation and quality-control criteria. Their labeled-window distributions were strongly concentrated in the L class because traffic-dependent timing produced substantially longer manual-driving intervals. Consequently, the reported mean LOEO metrics do not quantify generalization to these two sessions and should be interpreted only as session-level generalization across the seven retained unseen sessions. The two highly skewed sessions also illustrate the session-level variability introduced by real-world traffic and road conditions, which should be considered in the design and evaluation of future on-road driver-state studies.

We also emphasize that the choice of dataset partitioning strategy should be driven by the underlying data structure and the intended application. No single evaluation metric can fully characterize classifier performance, and a meaningful assessment often requires reporting multiple metrics [69]. Moreover, in the presence of class imbalance common in physiological data, accuracy alone may be misleading. It is essential to incorporate metrics that account for both false positives and false negatives [101]. In our experiments, we report accuracy, precision, recall, F1-score, and Cohen’s kappa to provide a balanced view of model performance.

### 8.7. Implications of LOEO Evaluation in Low-Session Target-Driver Datasets

Users 2, 3, and 4 were also examined as possible target drivers in preliminary LOEO trials. However, these analyses did not provide a fair or stable evaluation of target-driver generalization. The main reason is that these participants contributed only 4, 3, and 2 valid sessions, respectively. Under a LOEO protocol, this means that each held-out fold represents a large and highly specific fraction of the available data. As a result, the models trained on the remaining sessions were unable to predict the held-out session in a reliable way, and the fold-level results were dominated by session-specific class distributions rather than by stable predictive patterns.

For this reason, we did not report Users 2–4 as equivalent target-driver evaluations. Including those results would have produced estimates that were not directly comparable with the seven-fold User 1 evaluation and could have led to misleading conclusions about intra-subject performance. Instead, these users were used as external-user data in the main comparison, while User 1 was retained as the primary target driver because this participant provided the largest number of valid repeated sessions.

This observation provides an important methodological lesson for personalized DMS research. LOEO validation requires not only a sufficient number of labeled windows but also a sufficiently large number of independent driving experiences from the same driver. Overlapping windows increase the number of training samples, but they do not compensate for a low number of independent sessions. Robust target-driver validation therefore requires denser longitudinal data collection, with many more valid sessions per participant, to reduce fold instability and improve the reliability of session-level generalization estimates.

The amount of individualized data required for a subject-specific model depends on the architecture, the adaptation strategy, the type of signal considered, the temporal segmentation, and the stability of the labels. In the present study, personalization used 2158 labeled 30 s image windows from User 1, generated from wrist-derived physiological and motion signals, including BVP, EDA, HR, TEMP, and triaxial acceleration, across nine valid sessions; session-level generalization was assessed with seven retained LOEO folds. Other intra-subject and subject-dependent studies use different temporal scales. In wearable affect recognition, personalized models were trained from approximately 36 min of physiological recordings per subject, resampled at 700 Hz, segmented into 64-point windows with 50% overlap, and split in temporal order using 70% of each affective state for training, 15% for validation, and 15% for testing [26]. In ECG-based cognitive-load classification for automated driving, the within-subject evaluation used 20 min of ECG per participant, corresponding to 120,000 raw samples per ECG channel before preprocessing, with 60 s ECG windows and a 10-fold continuous split in which nine folds were used for training and one for testing [65]. In real-road driver-sleepiness detection, subject-dependent adaptation used ECG and electrooculography (EOG) features extracted from approximately 90 min driving sessions, summarized in 2 min feature windows, and evaluated settings where 10% or 30% of the target driver’s data were included in training [61]. These examples show that practical personalization is defined by the amount of temporal coverage, the number of usable windows, the signal modality, and the proportion of target-driver data required for adaptation. For deployment, the desirable direction is to reduce labeled target-driver calibration through transfer learning, semi-supervised learning, incremental updating, federated learning, and personalized fine-tuning [102]. Active learning provides a complementary sample-efficient strategy by combining model uncertainty and sample representativeness to prioritize the target-driver samples that would be most informative to annotate [103]. In the context of the present study, this approach could reduce the manual annotation effort required in subsequent personalized data collection campaigns, although it would still require candidate intervals for which the protocol context and a reliable ground-truth label remain available.

### 8.8. Data Protection Regulations

DMSs inherently rely on human data, including facial images, body posture, physiological signals, and behavioral patterns for model development and evaluation. According to the General Data Protection Regulation (GDPR) established by the European Union [104], data controllers are required to minimize the collection and processing of personal data whenever possible. In the context of DMSs, most input modalities qualify as personal or sensitive data, and aggregating such data across users may conflict with the GDPR’s data minimization principle. Prior studies have emphasized the need for strong privacy protections and clearly defined accountability in the design of these systems [105,106,107], particularly when sensitive data such as emotion recognition or physiological signals are involved [108,109].

In this paper, we observed that the target-driver-only configuration achieved higher performance than configurations using the available external-user data in the evaluated User 1 setting. This result suggests that personalization may be useful for reducing cross-user data dependencies in this type of DMS pipeline. From a data-protection perspective, target-driver models may also be attractive because they can reduce the need to aggregate identifiable physiological and behavioral data across users. However, this should be interpreted as a privacy-relevant design implication, not as proof that personalized DMS are universally superior or automatically GDPR-compliant. These findings are consistent with recent work advocating for personalized DMSs approaches as a means to reduce data exposure risks [110,111].

Although GDPR compliance extends beyond model training to aspects such as data storage, user consent, and algorithmic explainability, our study specifically emphasizes the minimization of cross-user data dependencies from both legal and ethical perspectives. Importantly, developing user-specific models does not preclude the incorporation of generalized knowledge derived from multi-user data. Techniques such as federated learning, representation transfer, and knowledge distillation can enable population-level insight while preserving the privacy of individual drivers [112,113].

Moreover, our research, which involves modeling drivers based on physiological data and video recordings, falls under the scope of the European Union’s Artificial Intelligence (AI) Act [114], particularly its provisions concerning high-risk AI systems in safety-critical domains such as driving automation. This legislation mandates comprehensive risk assessments, transparency in data processing, and robust safeguards for personal data. Our use of informed consent and data minimization is aligned with these requirements and supports the responsible development of DMS technologies under ethical and regulatory constraints.

### 8.9. Limitations

The primary boundary condition of this study is that the performance evidence is target-driver specific. Under the intra-subject modeling strategy adopted in this work, the model is learned from repeated observations of a selected target driver, and the resulting LOEO metrics must therefore be interpreted within that personalized setting. The reported performance figures should not be read as an estimate of model behavior for a general driving population, nor as evidence that the same numerical results would necessarily be obtained for other drivers. This does not undermine the rationale of intra-subject modeling: any driver could, in principle, be defined as the target driver, and the same methodological pipeline could be applied using repeated data from that individual. However, the resulting model behavior and performance would need to be evaluated separately for each new target driver.

The participant profile further constrains the scope of the empirical evidence. The study involved four male participants with prior experience operating the instrumented vehicle and the OpenPilot-based SAE Level 2 system. This profile is coherent with the operational requirements of the on-road experiment and with the intra-subject objective of the study, but it must be considered when interpreting the results. Crucially, within the proposed target-driver approach, the model is not intended to capture gender- or population-level patterns; it is trained exclusively from repeated data collected from the specific driver being modeled. In principle, therefore, any driver could serve as the target driver—regardless of age, gender, driving experience, physiological profile, or familiarity with driving automation—provided that sufficient repeated data are available for that individual. Nevertheless, because the empirical evaluation was conducted within a limited and demographically homogeneous participant group, the reported results should not be interpreted as evidence that equivalent model behavior or performance would necessarily be obtained for drivers with different demographic or operational characteristics. The limited age range is therefore an important constraint because age may influence physiological baselines, movement patterns, familiarity with driving automation, and session-to-session variability.

The operational nature of the labels constitutes a further boundary of the work. LL, L, H, and HH are derived from predefined protocol phases and scenario groups and are useful for evaluating whether the wearable-signal pipeline can discriminate between experimentally defined operational conditions. They should not, however, be interpreted as validated measurements of workload, stress, distraction, drowsiness, trust, or situation awareness.

The current level of classification performance also limits the practical interpretation of the system. The target-driver-only LOEO configuration achieved moderate results, with 60% accuracy, 52% macro-F1, and Cohen’s kappa of 0.43. These figures are reasonable for an initial real-world feasibility study using non-intrusive wearable signals and support the feasibility of the proposed personalized approach under the evaluated conditions. However, establishing its readiness for deployment as a DMS will require further validation.

Taken together, these limitations delimit the claims advanced in this study. The data acquisition procedure, experimental protocol, labeling process, and modeling pipeline were all designed with the target-driver setting as the central objective. The selected target driver should accordingly not be understood as a representative member of a broader population, nor as a driver chosen because of any expected model advantage. Rather, the target driver was selected within the available participant pool according to practical and operational criteria: the capacity to attend repeated on-road sessions, the availability of sufficient longitudinal data for intra-subject modeling, and non-involvement in the design or analysis of the study. This selection criterion is consistent with the methodological objective of evaluating a target-driver pipeline, but must be considered when interpreting the empirical results. The same experimental and modeling procedure could be applied by designating a different target driver and collecting repeated data from that individual. The results therefore support the feasibility of target-driver intra-subject modeling under the specific real-world conditions evaluated here, while broader evidence across drivers, demographic profiles, and deployment scenarios remains to be established.

## 9. Conclusions

This study examined the feasibility of personalized classification of scenario-derived operational driver-state classes based on non-intrusive wrist-derived physiological and motion signals under real-world SAE Level 2 automated driving conditions. The results provide initial evidence that target-driver intra-subject modeling can support this classification task under the specific conditions evaluated. In the evaluated setting, target-driver-only training yielded higher mean session-level classification performance than the configurations incorporating the available external-user data, supporting the personalization hypothesis within the evaluated target-driver setting. Specifically, the target-driver-only configuration achieved approximately 60% accuracy and Cohen’s kappa of 0.43 in the primary LOEO evaluation, compared with 50% accuracy and (κ=0.30) for the external-user-only configuration and 54% accuracy and (κ=0.36) for the mixed target/external-user configuration.

The results provide initial feasibility evidence that wrist-derived physiological and motion signals contain information relevant to distinguishing the scenario-derived operational driver-state classes in the evaluated real-world automated driving context. At the class level, the LL baseline class was the most reliably identified, with 88.4% one-vs.-rest accuracy and 70% F1-score, whereas H and HH were more difficult to separate, both reaching 44% F1-score. This evidence should be read as a feasibility result, not as the validation of a deployment-ready DMS. The current model is best understood as an exploratory personalized sensing component that could contribute to a broader multimodal driver-monitoring architecture once extended and validated at scale.

A second conclusion is that real-world experimentation is a necessary complement to simulator-based research. Controlled environments remain essential for repeatable and safe experimentation, but public-road experiments expose constraints that directly affect DMS development: traffic-dependent scenario execution, safety-driven interruptions, sensor-synchronization demands, missing data streams, and session-to-session variability. These are features of the operational reality in which future DMSs must function, and they cannot be fully characterized through laboratory or simulator studies alone.

Finally, the work underscores the importance of calibrating the strength of the conclusions to the experimental design. The results are specific to the target-driver evaluation, the available repeated sessions, and the operational labels defined in the protocol. They should not be interpreted as population-level evidence, nor as proof that intra-subject models are universally superior to inter-subject approaches. In this study, the target driver is the individual for whom repeated data were available and for whom the personalized model was trained, but the target-driver role is methodological rather than person-specific. Any driver could occupy this role, provided that sufficient repeated data were collected from that individual and the same experimental and modeling procedure were applied. The current participant profile also limits demographic interpretation, particularly regarding age diversity; broader validation will require additional target drivers from wider demographic ranges and repeated sessions for each individual. The contribution of this work is therefore not to generalize the reported numerical results to the broader driving population but to provide an initial real-world demonstration that personalized wearable-signal modeling is a technically feasible and methodologically principled research direction for future DMS development.

## 10. Future Work

A primary direction for future work is the integration of personalized driver-state models into operational vehicles for evaluation under real-time constraints, maintaining a non-intrusive data acquisition setup throughout. As deployment readiness was beyond the scope of the present study, this future evaluation will investigate the requirements associated with a deployment-ready DMS, including real-time inference latency, model updating strategies, and robustness under varied operational conditions. All experimentation will continue to adhere strictly to ethical guidelines and data protection regulations, ensuring responsible handling of personal data. Advances in DMS reliability could strengthen driver-state monitoring under sustained automation and, in turn, contribute to the broader safety objectives of ADS development.

The modeling pipeline evaluated in this study was limited to Empatica E4 wrist-derived signals; future research will incorporate the recorded visual streams into the modeling stage. In particular, subsequent work will explore the fusion of physiological and motion signals with camera-based behavioral features within a shared feature extraction pipeline, with the aim of building a more robust multimodal architecture for personalized driver-state sensing.

Label scarcity remains a key open challenge. Future efforts will investigate synthetic data generation and incremental learning methods to support real-time personalization without full model retraining. Broader research directions include characterizing the temporal stability of driver-specific physiological and motion patterns, optimizing multimodal input configurations, and evaluating personalization strategies across diverse user populations. Future research will also evaluate robustness through controlled experiments involving representative motion artifacts, signal dropout, degraded sensor contact, and other abnormal sensing conditions.

## Figures and Tables

**Table 1:** Summary of the multi-sensor acquisition setup.

Sensor/System	Location	Recorded Stream	Role
Comma Three front camera	Windshield	Road scene video, 10 fps	Protocol verification
Comma Three driver camera	Cabin-facing	Infrared face video, 10 fps	Protocol verification
Intel RealSense D435i	Passenger-side windshield	RGB, infrared, depth, and IMU	Protocol verification
Empatica E4	Driver wrist	BVP, EDA, TEMP, HR, and triaxial wrist acceleration	Model input
Vehicle/OpenPilot logs	Vehicle/OpenPilot system	Vehicle and automation logs	Protocol verification

**Table 2:** Recorded sessions, valid supervised sessions, and role of each participant.

Participant	Recorded Sessions	Valid Sessions	Role
User 1	26	9	Target
User 2	5	4	External
User 3	4	3	External
User 4	4	2	External

**Table 3:** Mapping between protocol phases, scenario groups, and operational labels.

Label	Source Interval	Included Phases or Scenarios
LL	Parked baseline	Initial baseline recording
L	Manual driving	Driver-controlled operation before DAS engagement
H	DAS engaged	Head up, head down, and dashboard gaze scenarios 3–12
HH	DAS engaged	Lane change and sudden startle scenarios 1–2 and 13–16

**Table 4:** Number of labeled samples per operational class and participant.

Class	User 1	User 2	User 3	User 4	Total
LL	429	259	192	137	1017
L	776	263	263	246	1548
H	513	368	133	202	1216
HH	440	398	237	169	1244
Total	2158	1288	825	754	5025

**Table 5:** Percentage distribution of labeled samples per operational class and participant. Percentages are calculated relative to the total number of samples for each participant.

Class	User 1	User 2	User 3	User 4
LL	20	20	23	18
L	36	20	31	32
H	23	29	16	27
HH	20	31	29	22

**Table 6:** Individual performance metrics for the four scenario-derived operational driver-state classes under the LOEO evaluation.

Class	A (%)	P (%)	R (%)	F1 (%)	κ
LL	88.40	72.00	68.00	70.00	0.52
L	69.61	57.00	64.00	60.00	0.36
H	73.55	45.00	44.00	44.00	0.27
HH	78.61	47.00	40.00	44.00	0.30

**Table 7:** Overall accuracy and Cohen’s kappa under different wearable-signal input configurations. “All wearable signals” includes BVP, EDA, HR, TEMP, and the three wrist-acceleration axes (ACCEL_X, ACCEL_Y, ACCEL_Z). “Physiological signals only” includes BVP, EDA, HR, and TEMP, excluding wrist acceleration.

Input Configuration	A (%)	κ
All wearable signals (BVP + EDA + HR + TEMP + ACCEL)	64.20	0.506
Acceleration only (ACCEL_X + ACCEL_Y + ACCEL_Z)	52.90	0.388
BVP only	35.92	0.127
HR only	35.60	0.109
EDA only	34.48	0.104
TEMP only	34.20	0.009
Physiological signals only (BVP + EDA + HR + TEMP)	58.20	0.483
All wearable signals except BVP	64.10	0.509
All wearable signals except HR	65.80	0.525
All wearable signals except EDA	63.10	0.495
All wearable signals except TEMP	64.90	0.521

**Table 8:** Classification accuracy for different signal-to-image encoding techniques. Bold indicates the best-performing encoding technique.

Encoding Technique	Accuracy (%)
MTF (4 states)	57.90
MTF (128 states)	53.80
RP (Binary)	56.50
**RP (Continuous)**	**64.20**
GASF	54.90
GADF	45.40

**Table 9:** Performance comparison between Leave-One-Experience-Out (LOEO) evaluation and random sampling (RS). Metrics include accuracy (A), precision (P), recall (R), F1-score, and Cohen’s kappa (κ). Bold identifies evaluation-group headings and aggregate summary statistics.

Evaluation	A (%)	P (%)	R (%)	F1 (%)	κ
**Leave-One-Experience-Out (LOEO). **					
Exp 1	56.43	57.32	59.51	55.87	0.4215
Exp 2	42.23	45.62	45.07	41.89	0.2388
Exp 3	83.96	47.53	42.19	44.70	0.6881
Exp 6	50.55	63.20	47.76	47.34	0.3181
Exp 7	56.99	52.74	51.86	50.73	0.3621
Exp 8	62.64	64.06	60.60	60.92	0.4922
Exp 9	62.41	65.66	63.44	63.07	0.4943
**Mean**	**59.32**	**56.59**	**52.92**	**52.07**	**0.4307**
**Std. Dev.**	**12.98**	**8.16**	**8.34**	**8.12**	**0.15**
**Random Sampling (RS)**					
**Mean**	**60.20**	**59.30**	**56.20**	**55.30**	**0.486**
**Std. Dev.**	**6.06**	**3.97**	**3.43**	**3.06**	**0.11**

**Table 10:** Offline inference latency of the proposed wearable-signal classification pipeline. Values are reported in milliseconds per 30 s multimodal window. Bold indicates the total latency of the complete processing pipeline.

Pipeline Stage	Mean	P50	P90	P95	P99
Signal-to-image conversion	4.493	4.695	5.309	5.481	5.831
ResNet-50 feature extraction	8.872	9.491	10.692	11.026	11.769
Feature fusion	0.098	0.096	0.118	0.125	0.147
PCA projection	1.214	1.205	1.440	1.565	1.998
MLP classifier	0.333	0.339	0.412	0.431	0.488
**Total pipeline**	**15.020**	**15.870**	**17.629**	**18.105**	**19.129**

## Data Availability

The data supporting the findings of this study are not publicly available due to GDPR requirements and the conditions of the participants’ informed consent. The dataset includes physiological signals, video recordings, and potentially identifiable behavioral data. De-identified or aggregated data may be made available from the corresponding author upon reasonable request, subject to institutional approval and data-sharing agreements. The code developed for this study, including the scripts for signal processing, signal-to-image transformation, feature extraction, model training, and evaluation, is available in a GitHub repository: https://github.com/raulfernmat/intra-subject-dms.

## Abbreviations

The following abbreviations are used in this manuscript:

ACC	Adaptive Cruise Control
ACCEL	Wrist Acceleration
ADASs	Advanced Driver Assistance Systems
ADS	Automated Driving System
ALC	Automated Lane Centering
BVP	Blood Volume Pulse
CAN	Controller Area Network
CNN	Convolutional Neural Network
DAS	Driving Automation System
DDT	Dynamic Driving Task
DMS	Driver Monitoring System
ECG	Electrocardiography
EDA	Electrodermal Activity
EEG	Electroencephalography
GADF	Gramian Angular Difference Field
GAF	Gramian Angular Field
GASF	Gramian Angular Summation Field
GDPR	General Data Protection Regulation
H	High
HH	High-High
HR	Heart Rate
IMU	Inertial Measurement Unit
L	Low
LL	Low-Low
LOEO	Leave-One-Experience-Out
MTF	Markov Transition Field
OEDR	Object and Event Detection and Response
ODD	Operational Design Domain
PCA	Principal Component Analysis
PHEV	Plug-in Hybrid Electric Vehicle
PPG	Photoplethysmography
ReLU	Rectified Linear Unit
RGB	Red–Green–Blue
RP	Recurrence Plot
rPPG	Remote Photoplethysmography
RS	Random Sampling
SAE	Society of Automotive Engineers
TEMP	Skin Temperature
t-SNE	t-distributed Stochastic Neighbor Embedding
UMAP	Uniform Manifold Approximation and Projection
UWS	University of the West of Scotland
